# Adjective position and referential efficiency in American Sign Language: Effects of adjective semantics, sign type and age of sign exposure

**DOI:** 10.1016/j.jml.2022.104348

**Published:** 2022-06-09

**Authors:** Paula Rubio-Fernandez, Anne Wienholz, Carey M. Ballard, Simon Kirby, Amy M. Lieberman

**Affiliations:** a University of Oslo, Norway; b University of Hamburg, Germany; c Boston University, United States; d University of Edinburgh, United Kingdom

**Keywords:** American Sign Language, Referential communication, Adjectives, Prenominal vs postnominal position, Efficiency

## Abstract

Previous research has pointed at communicative efficiency as a possible constraint on language structure. Here we investigated adjective position in American Sign Language (ASL), a language with relatively flexible word order, to test the incremental efficiency hypothesis, according to which both speakers and signers try to produce efficient referential expressions that are sensitive to the word order of their languages. The results of three experiments using a standard referential communication task confirmed that deaf ASL signers tend to produce absolute adjectives, such as color or material, in prenominal position, while scalar adjectives tend to be produced in prenominal position when expressed as lexical signs, but in postnominal position when expressed as classifiers. Age of ASL exposure also had an effect on referential choice, with early-exposed signers producing more classifiers than late-exposed signers, in some cases. Overall, our results suggest that linguistic, pragmatic and developmental factors affect referential choice in ASL, supporting the hypothesis that communicative efficiency is an important factor in shaping language structure and use.

## Introduction

A central question for linguistics is where constraints on variation in language structure come from. An influential account treats word order constraints as a result of languages adapting to the communicative needs of interlocutors (Gibson et al., 2019). In other words, efficiency pressures on language use may constrain language structure. In this paper, we construed communicative efficiency as the interplay between language structure and language use, and investigated the order of adjectives and nouns in a language with relatively flexible word order, American Sign Language (ASL). Specifically, we used different adjective classes to see whether ASL signers exploit their flexible noun phrase structure for communicative efficiency.

Scalar adjectives, such as size or width, depend on the interpretation of the noun. For example, a ‘big beetle’ is a very different size from a ‘big dog’. On the other hand, the interpretation of absolute adjectives such as color or material is far less dependent on the noun (compare ‘brown beetle’ and ‘brown dog’). If languages respond adaptively to efficiency pressures, then in a language with flexible noun phrase structure, we might expect scalar adjectives to appear after the noun upon which their interpretation relies (or show more variability), but color and material adjectives to appear before the noun. To foreshadow our results, we observed that ASL adjective ordering shows evidence of being able to respond flexibly to the communicative needs of the signers, suggesting that communicative efficiency is an important factor in shaping language structure and use.

### Adjective position and efficiency

Recent psycholinguistic studies have shown that adjective position affects the use and processing of color adjectives. Rubio-Fernandez et al. (2020; see also Rubio-Fernandez, 2016, 2019, 2021; Wu & Gibson, 2021; Kachakeche et al., 2021) observed that English speakers produced more redundant color adjectives than Spanish speakers (e.g., referring to a single star in a display of shapes as ‘the blue star’). This cross-linguistic difference supported the *incremental efficiency hypothesis*, according to which redundant color adjectives are more efficient in prenominal position because they guide the listener’s visual search for a referent, whereas in languages with postnominal modification (such as Spanish), the listener’s visual search is guided by the noun (for eye-tracking evidence, see Rubio-Fernandez & Jara-Ettinger, 2020). Offering further support to the incremental efficiency hypothesis, both English and Spanish speakers produced higher and comparable rates of redundant modification in denser displays of shapes where it was harder to identify a referent and both prenominal and postnominal color adjectives could facilitate visual search (Rubio-Fernandez et al., 2020).

In another recent test of the incremental efficiency hypothesis, Jara-Ettinger and Rubio-Fernandez (2021) observed that the results of a reference rating task (in which participants had to rate on a 1–7 scale how likely they were to use a referential expression) strongly correlated with the pattern of response times in an eye-tracking task using the same materials. Thus, participants’ ratings did not only reveal a preference for color descriptions (e.g., ‘the red chair’) over material descriptions (e.g., ‘the leather chair’): the ratings revealed that participants were more likely to use those descriptions that led to faster identification times in a given display – whether they were color or material.

The incremental efficiency hypothesis has been tested on the production and comprehension of several spoken languages, but not on sign languages. In a recent eye-tracking study on ASL, in which both pre- and post-nominal adjectives are possible, Wienholz and Lieberman (2019) showed that the word order of color adjectives and nouns only played a minor role on adult signers’ visual search for referents in a comprehension task. However, there are unique features related to visual search in studies of sign language sentence processing. Whereas in visual-world spoken language studies comprehension is cross-modal, in sign language the signer and the potential referents are integrated in the same visual display. As a result, signers must strategically allocate visual attention in order to perceive both the linguistic input and the referent in sequence (Lieberman et al., 2018; Wienholz & Lieberman, 2019).

Signers are adept at efficiently allocating their visual attention from a young age (Lieberman et al., 2014). In Wienholz and Lieberman’s (2019) study, if the adjective or noun provided sufficient information to identify the target early in the sentence, signers would shift gaze to the target during a prosodic pause mid-sentence, and then shift gaze back to the signer to perceive the rest of the sentence. In contrast, if there was insufficient information early in the sentence to identify the referent, signers would maintain gaze to the signer until the end of the sentence, and only then shift gaze away from the linguistic input. Thus, allocation of attention and strategic gaze shifting are additional factors that determine gaze patterns in visual search during sign comprehension, and which are related to efficient language processing.

Further research is therefore needed to understand the role of adjective position in sign language production and comprehension. In the case of ASL, fundamental questions remain about word order preferences. It is unclear whether prenominal or postnominal adjectives are used predominately. Likewise, it has yet to be tested whether adjective position varies depending on the semantics of the adjective (e.g., absolute vs scalar) or the type of sign used to express a scalar property (i.e., classifier vs lexical sign). However, these research questions are not only important for our understanding of ASL, but also as a test of referential efficiency in human communication. We therefore addressed these questions in three reference production experiments with deaf ASL signers.

### Adjective position in ASL

In common with all languages, linguistic structure in ASL is organized at multiple levels (Klima & Bellugi, 1979; Wilbur, 1979). ASL sentences use a basic subject-verb-object (SVO) word order (Fischer, 1975), however variations in word order can occur as the result of grammatical variations including topicalization, pro-drop, and subject copy (Kegl & Wilbur, 1976; Padden, 1998). Acquisition of word order in ASL has primarily been studied with regard to subject, verb, and object position. Children acquiring ASL initially use more variable word order than adults (Hoffmeister, 1978; Schick et al., 2002), but by the age of three years their word order is more likely to be canonical (Berk, 2003). Late learners of ASL who are acquiring ASL as a first language in adolescence show a similar trajectory as children—moving from non-canonical to canonical word order—however this process may be protracted in this population (Cheng & Mayberry, 2019).

Within this general constituent order, ASL generally allows for adjectives to occur in both pre- and postnominal positions: an ASL sentence such as PRO-3 WANT RED BOOK^1^ (‘He wants the red book’) can also be produced as PRO-3 WANT BOOK RED. The flexible nature of adjective position specifically and word order more generally makes ASL an ideal test case for hypotheses around referential efficiency in language production. Although referential efficiency has not previously been tested in ASL, several factors have been proposed for signers choice of adjective position. Earlier characterizations point to particular characteristics of the signer. For example, older signers may use more prenominal adjectives (Gee & Kegl, 1983), which suggests that language usage may have changed over time (perhaps influenced by the surrounding spoken language) to favor prenominal adjectives. In grammaticality judgments, Boster (2013) found that while some signers prefer noun-adjective and others prefer adjective-noun order, signers generally note that their non-preferred order is at least marginally acceptable, provided that the noun and adjective are adjacent in the utterance.

Some researchers have argued that adjective position depends on their syntactic function within the noun phrase. Critically, however, identifying the lexical category of signs is not always transparent. In particular, noun modifiers that occur postnominally can, in some cases, be interpreted equally well as adjectives or predicates (Schwager & Zeshan, 2008). As there is no copula in ASL, a phrase such as BOY FUNNY could be interpreted as ‘the funny boy,’ but could also be interpreted as ‘the boy *is funny.*’ Padden (1998) includes criteria for defining adjectives such that they must be able to inflect for intensive aspect. Padden further explains that adjective-noun sequences cannot receive a clausal interpretation in ASL, so that adjectives that can only function as predicates must be produced in postnominal position.

MacLaughlin (1997) studied determiner phrases in ASL constructions. In her analysis of adjective position, she proposes that pre- and postnominal adjectives should be analyzed differently with respect to syntactic function (Loos, 2022). For example, prenominal adjectives in ASL are subject to ordering restrictions (e.g., BIG RED BALL is acceptable while RED BIG BALL is not), while postnominal adjectives are not. Second, prenominal adjectives can sometimes have idiosyncratic meaning, while postnominal adjectives have only compositional meaning. For example, in the ASL phrase OLD FRIEND, the adjective OLD can mean either ‘old in age’ or ‘long time’ friend; whereas in the phrase FRIEND OLD, the adjective OLD can only mean ‘old in age’. Finally, certain adjectives can only occur in prenominal position (e.g., FORMER, REAL/TRUE, BASIC) as part of the noun phrase. Other adjectives can only occur in postnominal position, including certain human propensity terms (e.g., FUNNY, HEALTHY, CONFUSED, UPSET) (Bernath, 2010; Padden, 1998; Valli & Lucas, 2000).

Loos (2022) suggests that adjective position may vary systematically by semantic class, such that core properties (e.g., color, dimension) and physical properties (e.g., consistency, temperature) may occur prenominally, while human propensities (i.e., physical and mental states) may occur postnominally. Loos provided ASL signers with signs (presented as still pictures) and instructed them to arrange them into as many grammatical ASL sentences as possible. The adjectives presented included property tokens (e.g., COLD, SOFT, DRY), corporeal states (e.g., SICK, HEALTHY) and human propensities (e.g., HAPPY, FUNNY). Participants had a strong preference for modification to occur in prenominal position, with property tokens occurring prenominally more often than tokens that occurred in predication. Participants also completed an acceptability judgment task using size and color signs in various sequences. In prenominal position, size-color and color-size were accepted, but participants rejected constructions with multiple postnominal property signs.

Another source of adjective placement variation is syntactic priming. Hall et al. (2015) investigated the likelihood that signers would produce a postnominal adjective based on whether the previous sentence they had seen also used a postnominal adjective. Participants were first shown an array of items containing sets of three objects in three different colors. They then saw a video of a signer instructing them to choose a specific object (e.g., CHOOSE BIRD GREEN). Following each prime trial, the participant then saw a new colored object that they had to describe to the experimenter using color and identity. The results showed that signers were more likely to produce an utterance using a postnominal adjective following postnominal primes than prenominal primes. This effect held across deaf native ASL signers, deaf late-exposed ASL signers, and hearing L2 signers, providing robust evidence that signers are susceptible to syntactic priming for adjective placement in ASL. However, the adjectives in this study were all color words, so it is unclear whether the effect generalizes to other types of adjectives.

### Classifier predicates in ASL

**Fig. 1.**
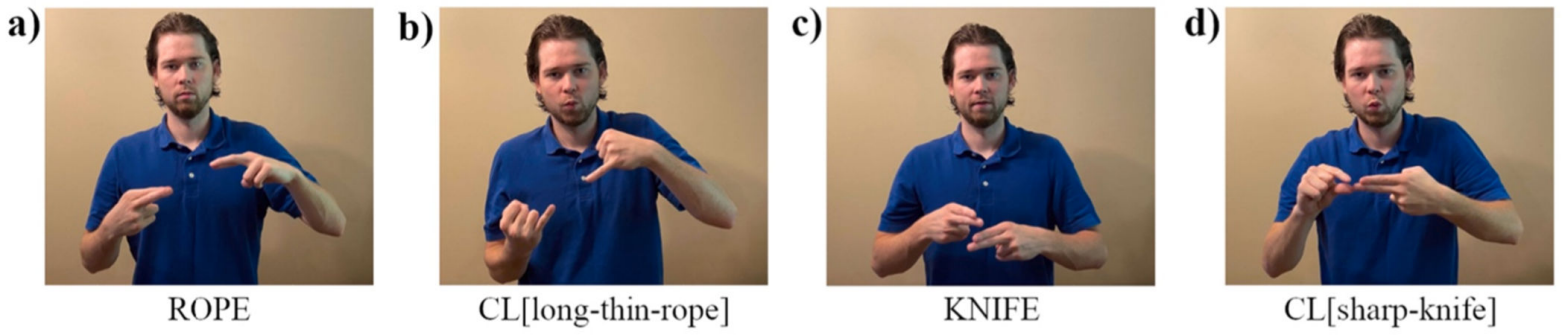
Adjectives expressed as classifier constructions: a) lexical sign for ROPE; b) classifier indicating size thickness; c) lexical sign for KNIFE; d) classifier construction indicating sharpness.

An additional feature of ASL may impact the way in which some modifiers are produced. The term ‘classifier’ is used to describe a range of constructions in sign languages that incorporate linguistic and gestural elements (Hodge & Johnston, 2014; Schembri et al., 2005), and are used to represent both entities and verbs of motion. Most relevant to the current study is a set of constructions that incorporate adjectival properties of an entity and are known as ‘size and shape specifiers,’ or ‘SASS’ constructions (Supalla, 1982). In SASS constructions, the handshape of a sign is modified to incorporate reference to the shape and/or dimensions of a referent noun. Supalla further subdivides SASS constructions into static SASSes and tracing SASSes depending on whether the hands move in a way that traces the size and shape of the referent (Supalla, 1986). In a later analysis, Zwitserlood (2003) argues that static and tracing SASSes function differently in NGT (Sign Language of the Netherlands): in static SASSes (also referred to as ‘entity classifiers’), the handshape represents the object; whereas in tracing SASSes both the handshape and the movement contribute to object representation. Zwitserlood suggests that tracing SASSes may be interpreted as adjectives, but also notes that adjectives and verbs may be difficult to distinguish in NGT. Notably, the handshapes used in SASS constructions are linguistically constrained and categorical, while the movement of tracing SASSes can express gradient features of an object’s size or shape (Emmorey & Herzig, 2003). For example, in order to depict a long, thin rope, a signer could produce a single classifier^2^ construction where the handshape depicts the thin size of the rope and the movement depicts the length of the rope. However, the lexical sign ROPE is not part of the classifier construction (see Fig. 1).

How might the use of SASS constructions impact adjective position? From a referential efficiency perspective, one possibility is that the most efficient construction is a single, multi-morphemic SASS construction that expresses both the entity and its dimensional properties. If this were the case, there may be no distinct noun and adjective. However, because SASSes only serve to specify the properties of the referent, they are not typically produced in isolation. Instead, signers typically identify the referent using a lexical sign (e.g., ROPE) and its features using a SASS construction (e.g., CLASSIFIER[long-thin-object]) (Fig. 1). We propose that this type of construction (i.e. referent-classifier) is far more efficient than the reverse (classifier-referent), as the classifier provides additional information about the properties of the referent, and these properties are best understood when the identity of the referent has already been specified. As an additional caveat, however, the presence of a classifier construction does not resolve the question of whether the expression should be interpreted as an adjective or a predicate; in fact it may be even more difficult to make this distinction than in expressions that rely on lexical adjectives. For example, the construction in 1(d) CL[sharp-knife] could be analyzed as an adjective (although there is also a lexical adjective that means ‘sharp’), but could also be analyzed as a predicate (i.e. ‘the knife is sharp’), which adheres to the SV constituent order of ASL.

Given their linguistic complexity, it is possible that individuals with delayed access to ASL may produce fewer classifier constructions than signers with early access to ASL. SASS classifiers show protracted development; children with native ASL exposure produce them with 80% accuracy by 5 to 8 years of age (Schick, 1990). Deaf individuals who acquired ASL as a first language after early childhood are less likely to use polymorphemic classifier constructions than native signers (Galvan, 1999; Newport, 1990). Similarly, adult second-language learners of sign languages find classifiers among the more difficult structures to acquire (Boers-Visker, 2021; Frederiksen & Mayberry, 2019), Conversely, late-exposed signers may produce some SASS-like morphemes even without regular exposure to adult models because they incorporate elements of gesture. Studies of deaf individuals who acquired ASL as a first language in adolescence have reported that these individuals begin producing classifiers after several months of exposure to ASL, sometimes as unanalyzed forms (Ferjan Ramírez et al., 2013; Morford, 2003). With regard to classifier comprehension, Beal-Alvarez (2014) found that children with deaf parents scored higher than children of hearing parents on items in the ASL Receptive Skills Test (ASL-RST; Enns et al., 2013) that relied on SASS constructions. This may arise from the fact that deaf parents use more classifiers in their spontaneous narratives than hearing parents (Morford & MacFarlane, 2003). Thus, we speculated that age of acquisition would influence the frequency and accuracy with which signers produced classifiers in the current task.

### Outline of the study

According to the incremental efficiency hypothesis, speakers and signers try to produce efficient referential expressions that are sensitive to the word order of their languages. However, this hypothesis has not been tested in sign languages, which may have different patterns of sign parsing and visual search during referential communication (see Wienholz & Lieberman, 2019; Wienholz et al., 2021) relative to the cross-modal processing observed in spoken languages (e.g., Rubio-Fernandez & Jara-Ettinger, 2020). In ASL, it has been observed that adjectives occur in both prenominal and postnominal position, and that adjective position is affected by age (Gee & Kegl, 1983; Klima & Bellugi, 1979; Padden, 1998) and by some syntactic constraints. However, to the best of our knowledge, adjective position in ASL has not been investigated from a referential efficiency perspective and, therefore, this is the aim of our study.

More specifically, we tested a prediction based on adjective semantics: we predicted that adjectives for absolute properties (such as color or material) would be produced in prenominal position because they can be interpreted independent of the noun. If the target referent can be identified from just the adjective, then an interlocutor could identify the target from this partial information. By contrast, from an incremental processing perspective, it would be more efficient to encode scalar adjectives (such as ‘big’ or ‘small’) in postnominal position because they are understood in relation to the noun they accompany. In addition, we investigated the degree to which scalar adjective position depends on the type of sign that is used to express a scalar property; namely, classifiers or lexical signs. Because handshapes in ASL classifier constructions reflect properties of the referent (such as its size and shape), and thus are also understood in relation to the referent noun, we predicted that they would be produced in postnominal position.

Contrary to these predictions, it is possible that adjective position in ASL is dependent on other factors (e.g., the naturalness of certain word orders; Goldin-Meadow et al., 2008, Culbertson et al., 2020), or that the communicatively efficient strategies documented in spoken languages have different counterparts in the visual-gestural modality of sign languages (Wienholz & Lieberman, 2019). As Caselli et al. (2022) have recently argued, what is efficient for communication in the visual-gestural modality may differ from the auditory-oral modality. Therefore, an investigation of communicative efficiency in referential communication must include both spoken and sign languages.

We investigated the hypotheses outlined above in three experiments using a standard referential communication task. Experiment 1 compared the production of color and scalar adjectives, while Experiment 2 compared the production of material and scalar adjectives. Experiment 3 tested the production of ‘big’ and ‘small’ in pop-out displays, in which the size of the target object was an absolute property (i. e., the target was the largest or the smallest object in the entire display). According to the incremental efficiency hypothesis, ASL signers should produce more prenominal size modification in pop-out displays than in non-pop-out displays because the size of the target is more visually salient and prenominal modification would be more efficient for processing. In addition to testing the incremental efficiency hypotheses, Experiments 2 and 3 also explored the effect of age of sign exposure (early-exposed vs late-exposed) on signers’ choice of referential expression. In particular, we wanted to investigate whether early-exposed signers produced more classifiers than late-exposed signers when expressing scalar properties.

## Experiment 1

### Data availability

All materials, data and analysis scripts for the three experiments in the study are publicly available in an Open Science Repository (see https://osf.io/xh6n5/).

### Methods

#### Participants

Participants were deaf adults (n = 33; 18 females, 11 males, and 4 did not report gender) who used ASL as their primary mode of communication. Participants ranged in age from 18 to 49 years (*M* = 27.5 years). Self-reported levels of hearing loss ranged from mild/moderate (n = 3) to severe/profound (n = 30). The majority of participants had deaf parents (n = 22); eight participants had hearing parents and three did not report parental hearing status. Most participants were exposed to ASL at birth (n = 19) or shortly after/before the age of three (n = 5), with the remainder exposed at various ages after birth (*M* = 16.6 years; range: 14–18 years). All participants reported using ASL as their primary form of communication at the time of the study.

#### Stimuli

**Fig. 2.**
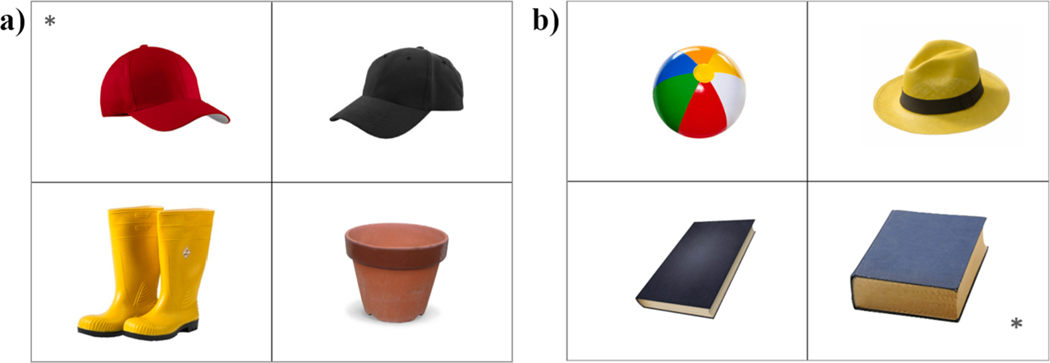
Sample items from (a) the color condition (elicited adjective: ‘red’) and (b) the scalar condition (elicited adjective: ‘thick’). Targets are marked with an asterisk.

The linguistic materials included eight color adjectives (black, blue, brown, green, orange, red, white, yellow) and eight scalar adjectives (big, narrow, short, small, tall, thick, thin, wide). For each adjective, three visual displays of four pictures were created resulting in 48 trials (see Supplemental Material A for the full list of stimuli). The pictures were photo-realistic images of objects on a white background and included a target, a contrast and two unrelated distractors (see Fig. 2 for sample displays). The target was marked with an asterisk and intended to elicit the relevant property (e.g., ‘red cap’ or ‘thick book’). The target objects were chosen to be common objects that were represented by a common, conventional ASL lexical sign. The contrast was an object of the same kind that did not possess the relevant property (e.g., a black cap or a thin book). Distractor objects were of a different kind than the target and the contrast and did not possess the relevant property. Target position was counterbalanced across the four quadrants in the display.

#### Procedure

Prior to the task, participants gave written signed consent and provided demographic information as well as information about their linguistic background via an online survey. The task was conducted by a deaf ASL signer. Participants were seated at a table in front of a 27-inch Apple iMac computer with a Sony camcorder placed behind the screen to capture participant responses from front view. The stimuli were presented using the Microsoft PowerPoint Desktop App. In a pre-recorded ASL instruction video, participants were asked to look at the visual displays and find the picture that is marked with an asterisk (i.e., the target). As in a standard referential communication task, the signers were instructed to refer to the target object in a way that would allow a future recipient to uniquely identify that object in the display.

Stimuli were presented in two blocks of 24 trials and participants could take a break between blocks if needed. Each block elicited only one type of adjective (color or scalar) in order to reduce possible carryover effects across trials (e.g., a color adjective priming prenominal modification in the following scalar trial). Within each block, trials were pseudorandomized with a maximum of two consecutive trials eliciting the same adjective. Block order was crossed in two lists of materials (color-scalar and scalar-color) and participants were randomly assigned to one of the two lists.

#### Data processing

Deaf native and hearing proficient ASL signers annotated participants’ responses using a pre-defined coding sheet including the following information: adjective position, produced adjective, and sign type. Regarding position, adjectives were coded as *prenominal* (i.e., preceding the noun), *postnominal* (i.e., following the noun), or *other*. The category *other* included instances where only the adjective was used (i.e. no noun was produced), either the adjective or the noun occurred twice (e.g., BALL BIG BALL or BIG BALL BIG), the adjective was incorporated in the noun using classifiers (i.e., adjective and noun are expressed as one sign; e.g., CLASSIFIER[big-ball]), a different adjective type was used (e.g., a color adjective instead of the intended scalar adjective), or no adjective was produced at all. In some cases, signers produced a different adjective of the same type (i.e., SMALL instead of SHORT) and those were coded according to the position of the adjective and retained for analysis. See Supplemental Material B for distribution of response types that were categorized as *other*.

In addition, for scalar adjectives, sign type was coded as *lexical sign*, *classifier* or *fingerspelled sign*. We used a definition of lexical signs as fixed forms that are stored in the mental lexicon and whose shape is not influenced by the modified object sign. In contrast, classifier signs are affected by the object sign they accompany and, thus, their handshape is modified to represent properties of the referent object. Finally, finger-spelled signs use the manual alphabet in which each letter of the written English word is represented with a unique handshape.

An additional deaf native ASL signer coded 25% of the data for reliability. To determine consistency among coders, a reliability analysis was performed using Cohen’s Kappa (Cohen, 1960) analyzing agreement on adjective position and sign type. Coders exhibit strong agreement for adjective position (κ =.90, 95% CI [.81,.99]) and sign type (κ =.80, 95% CI [.68,.91]).

In total, we elicited 1490 responses. Responses with adjective position coded as *other* (331 responses; 22% of the data; see Supplemental Material B for a breakdown of this category) and sign type coded as *fingerspelled* were excluded from analysis. Fingerspelled signs were removed because (1) they are neither lexical signs nor classifiers and (2) we could not determine whether participants used fingerspelling because they did not have a lexical sign/classifier for the respective adjective, or whether the experimental setting evoked the use of fingerspelling.

#### Analysis plan and predictions

Adjective position (prenominal vs postnominal) was analyzed using two logistic mixed-effects models that tested the incremental efficiency hypothesis, according to which adjectives for absolute properties (such as color) are more efficient in prenominal position, while scalar adjectives (such as size) are more efficient in postnominal position. The first model tested the effect of adjective type (color vs scalar) across all sign types (i.e., lexical signs and classifiers), while a second model focused on lexical signs. Our second prediction was that classifiers would be encoded in postnominal position, because classifier constructions refer to properties of the referent which must first be lexically specified. To investigate this hypothesis, we analyzed scalar adjective position using a logistic mixed-effects model testing for the effect of sign type (classifier vs lexical sign).

### Results

**Fig. 3.**
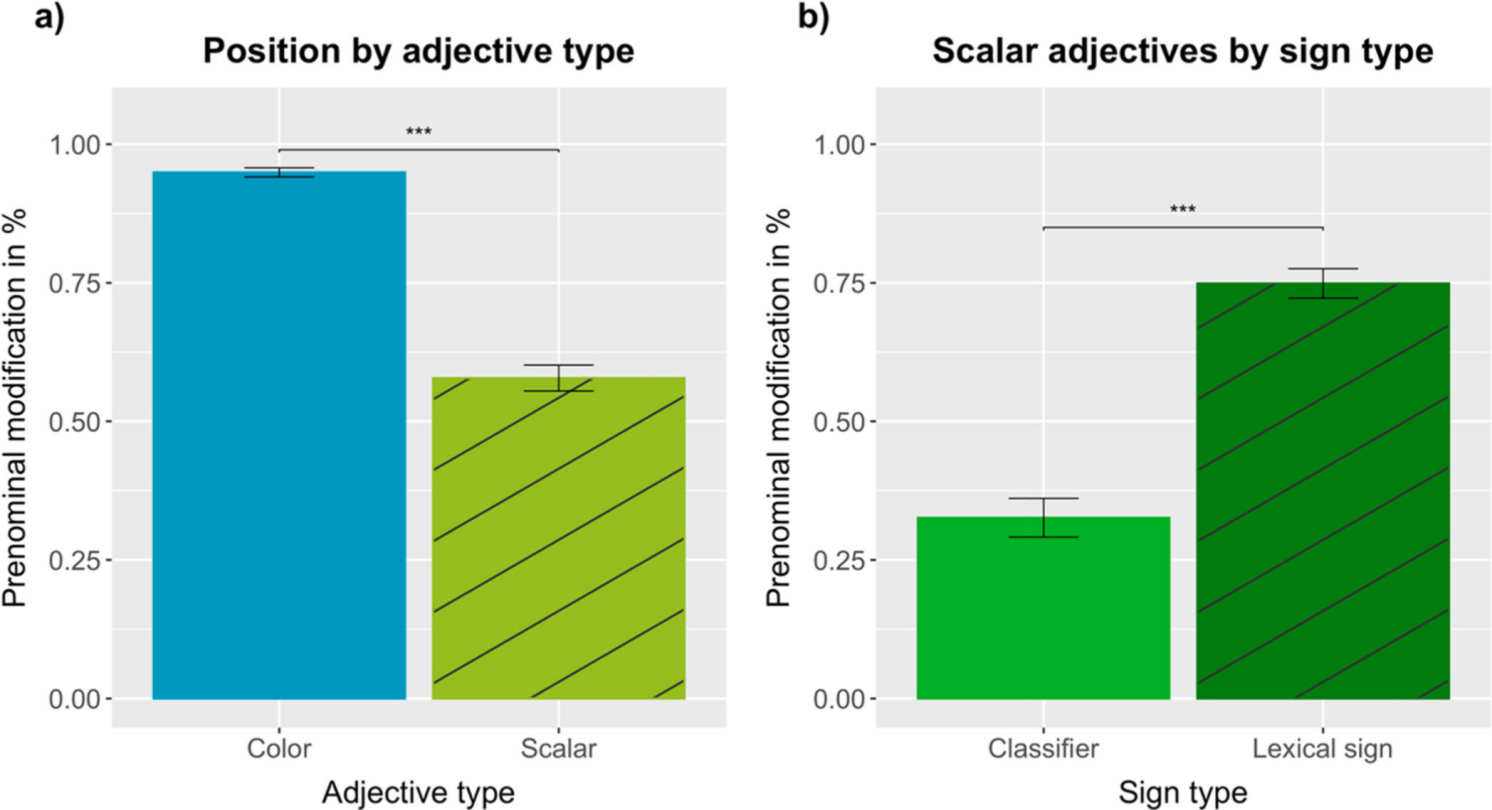
Percentage of (a) color and scalar adjectives and (b) classifiers and lexical signs (expressing scalar adjectives) produced in prenominal position.

**Fig. 4.**
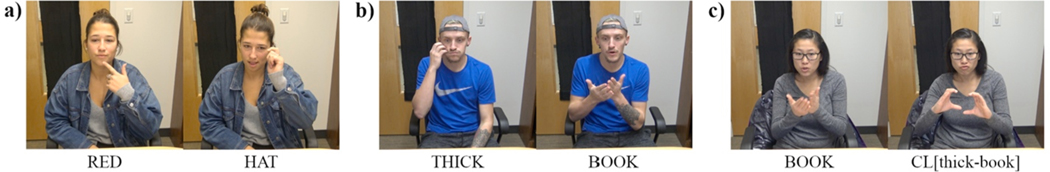
Video stills of participants producing the dominant word order for (a) color adjectives in prenominal position, and scalar adjectives in (b) prenominal and (c) postnominal position.

Adjective position was analyzed using a logistic mixed-effects model (prenominal = 1, postnominal = 0) with a fixed effect of adjective type (color vs scalar) and the maximal random effect structure for participants and items, including random intercepts for participants and items, plus random by-participant slopes for adjective type. Supporting our experimental predictions, there was a significant main effect of adjective type (*β* = −4.0235, SE = 0.6578, Z = −6.117, *p* < 0.0001), with color adjectives (*M* =.95, SD =.01) being produced in prenominal position significantly more often than scalar adjectives (*M* =.58, SD =.02) (see Fig. 3a; for video stills of produced word orders, see Fig. 4).

Since the difference between color and scalar adjectives could be driven by the use of classifiers in the scalar adjectives, we ran the same model on the subset of responses that were lexical signs (i.e., excluding classifier responses). As in the first model, we observed a significant main effect of adjective type (*β* = −3.0107, SE = 0.6448, Z = −4.669, *p* < 0.0001), confirming that lexical signs are encoded in prenominal position more frequently for color (*M* =.95, SD =.01) than for scalar properties (*M* =.75, SD =.03).

To investigate the effect of sign type on scalar adjective position, we analyzed the data from the scalar condition using a logistic mixed-effects model including a fixed effect of sign type (classifier vs lexical sign), random intercepts for participants and items, plus random by-participant slopes for sign type. Also supporting our experimental predictions, there was a significant main effect of sign type (*β* = 1.9963, SE = 0.3788, Z = 5.271, *p* < 0.0001), with lexical signs for scalar properties being produced in prenominal position (*M* =.75, SD =.03) significantly more often than the corresponding classifiers (*M* =.33, SD =.03) (see Fig. 3b).

While an analysis of the individual adjectives used in this study is beyond the scope of our investigation, descriptive statistics suggest that ASL signers have different encoding preferences for different scalar adjectives. For example, ‘big’ and ‘small’ were expressed as lexical signs over 75% of the time, while ‘tall’ and ‘short’ were expressed as classifiers just as frequently. In between were adjectives like ‘thin’ and ‘wide’, which were encoded as either type of sign around half the time (for a visualization of the production rates of lexical signs and classifiers for individual scalar adjectives, see the Supplemental Material C).

### Discussion of Experiment 1

The results of Experiment 1 confirmed our experimental predictions, offering support to the incremental efficiency hypothesis: ASL signers produced color adjectives almost exclusively in prenominal position, while scalar adjectives were signed both prenominally and postnominally. The latter effect was driven by the two types of signs commonly used in ASL to express scalar properties: lexical signs were more often produced in prenominal position, while classifiers were mostly produced in postnominal position. However, an analysis of sign position for just the lexical signs confirmed that lexical signs for color were encoded in prenominal position more often than lexical signs for scalar properties – as predicted by the incremental efficiency hypothesis.

## Experiment 2

The results of our first experiment were in line with our predictions. However, because color is a visually salient property and color adjectives have a special status in referential communication (e.g., they are used redundantly more often than other absolute adjectives, such as material or pattern; see Jara-Ettinger & Rubio-Fernandez, 2021; Kursat & Degen, 2021; Rubio-Fernandez, 2019; Sedivy, 2005; Tarenskeen et al., 2015), the strong preference to use color adjectives prenominally may not generalize to other absolute adjectives in ASL. To further test our hypotheses, we therefore conducted the same referential communication task in Experiment 2, but comparing material and scalar adjectives. In addition, we recruited more balanced groups of early-exposed and late-exposed ASL signers to investigate whether age of ASL exposure affects adjective position and/or the production of classifiers and lexical signs expressing scalar properties.

### Methods

#### Participants

Participants were deaf adults (n = 44; 24 females, 20 males) who used ASL as their primary mode of communication. Participants ranged in age from 19 to 50 years (*M* = 27.3 years). Self-reported hearing loss ranged from mild/moderate (n = 6) to severe/profound (n = 38). Most participants reported being identified as deaf at birth (n = 40), the rest reported being identified after birth (n = 4).

Because we were interested in the effects of age of exposure on adjective placements, we grouped participants based on their age of initial exposure to ASL. This grouping was based on participants’ answers to items in the background questionnaire that asked the age at which they were exposed to ASL and whether their primary caregiver communicated with them in ASL. Participants who reported that their age of ASL exposure was below the age of three years and that their primary caregiver communicated with them in ASL were categorized as *early-exposed signers*, whereas participants whose ASL exposure was above the age of three years were categorized as *late-exposed signers*. Using this categorization, our participant groups included 29 early-exposed signers and 15 late-exposed signers (*M* = 21.8 years; range: 14–31 years at exposure). We chose to group participants by the age at which they were exposed to ASL rather than by parental hearing status because the age of first language exposure is thought to be a better measure of early linguistic experience.^3^

#### Stimuli

**Fig. 5.**
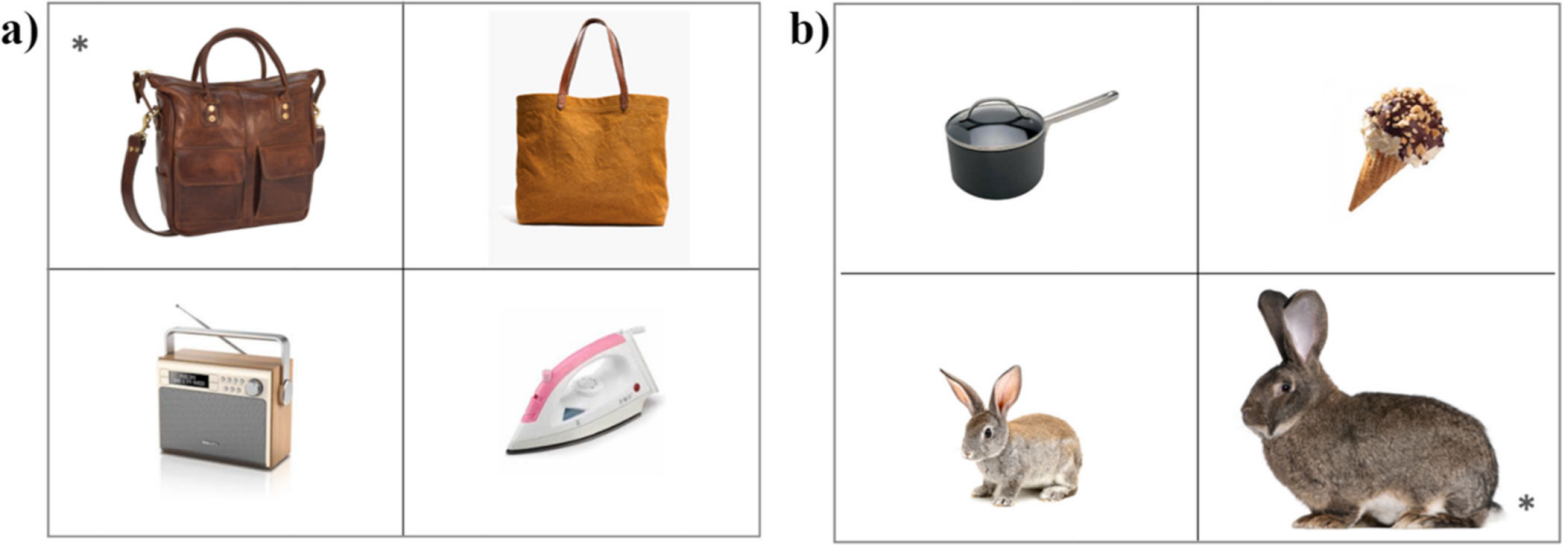
Sample items from (a) the material condition (elicited adjective: ‘leather’) and (b) the scalar condition (elicited adjective: ‘big’). Targets are marked with an asterisk.

As in Experiment 1, the linguistic materials included two lists of eight adjectives (see Supplemental Material D for the full list of stimuli). One list included material adjectives (cotton, glass, gold, leather, metal, paper, plastic, wooden) and the other included the same scalar adjectives as in Experiment 1 (big, narrow, short, small, tall, thick, thin, wide). The 24 visual displays from the scalar condition in Experiment 1 were used again in Experiment 2. However, 12 of the original pictures were replaced with more salient exemplars because they did not consistently elicit the target adjective in Experiment 1. Note that only the pictures were different: the scalar adjectives that we aimed to elicit were the same in the two experiments. In addition, three displays were created for each of the material adjectives, following the same specifications as with the scalar displays (see Fig. 5 for sample displays). Five of these pictures were replaced between two testing sessions because they were not consistently eliciting the target material adjectives. Again, only the pictures were replaced, not the target adjectives. The task design was the same as in Experiment 1: to avoid carryover effects, the 48 displays were distributed in two blocks by adjective type (material and scalar), which were presented in two block orders (material-scalar and scalar-material).

#### Procedure

The procedure for Experiment 2 was identical to Experiment 1.

#### Data processing

Data from Experiment 2 was processed following the same procedure as in Experiment 1. Again, Cohen’s Kappa (Cohen, 1960) was performed determining coders agreement on adjective position and sign type. Coders exhibit strong agreement for adjective position (κ =.85, 95% CI.79,.91]) and substantial agreement for sign type (κ =.77, 95% CI.69,.85]). Overall, 2022 responses were collected. However, 1028 (50.8%) responses were excluded from the analyses because adjective position was coded as *other* (996 responses) or sign type was coded as *fingerspelled* (32 responses).

#### Analysis plan and predictions

As in Experiment 1, adjective position (prenominal vs postnominal) was analyzed using two logistic mixed-effects models that tested the incremental efficiency hypothesis, according to which material adjectives are more efficient in prenominal position, while scalar adjectives are more efficient in postnominal position. The first model tested the effect of adjective type (material vs scalar) across all sign types, while the second model focused on lexical signs. Our second prediction was that classifiers would be encoded in postnominal position. To test this hypothesis, we analyzed scalar adjective position using a logistic mixed-effects model testing for the effect of sign type (classifier vs lexical sign). Finally, we explored the use of classifiers by early-exposed and late-exposed ASL signers, for which we analyzed the production of classifiers and lexical signs in the scalar condition using a logistic mixed-effects model testing for the effect of sign exposure (early-exposed vs late-exposed).

### Results

**Fig. 6.**
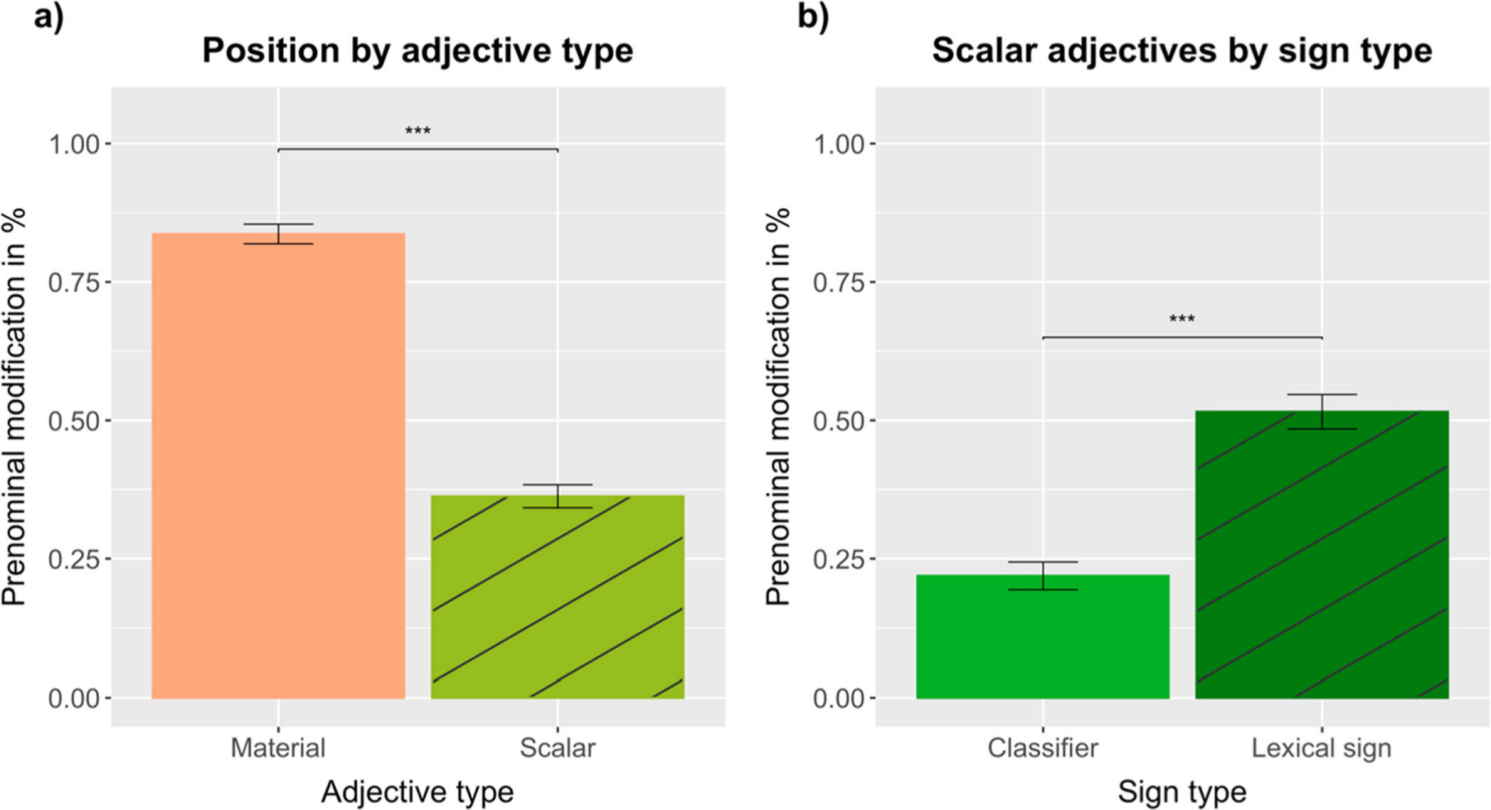
Percentage of (a) material and scalar adjectives and (b) classifiers and lexical signs (expressing scalar adjectives) produced in prenominal position.

**Fig. 7.**
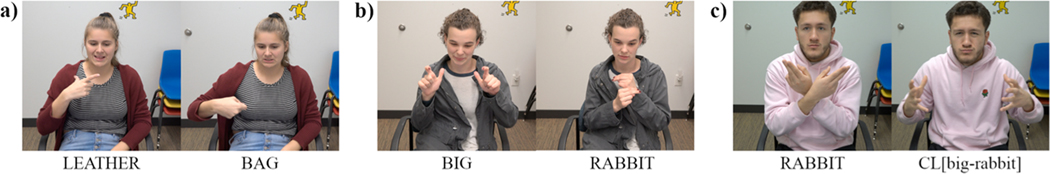
Video stills of participants producing the dominant word order for (a) material adjectives in prenominal position, and scalar adjectives in (b) prenominal and (c) postnominal position.

**Fig. 8.**
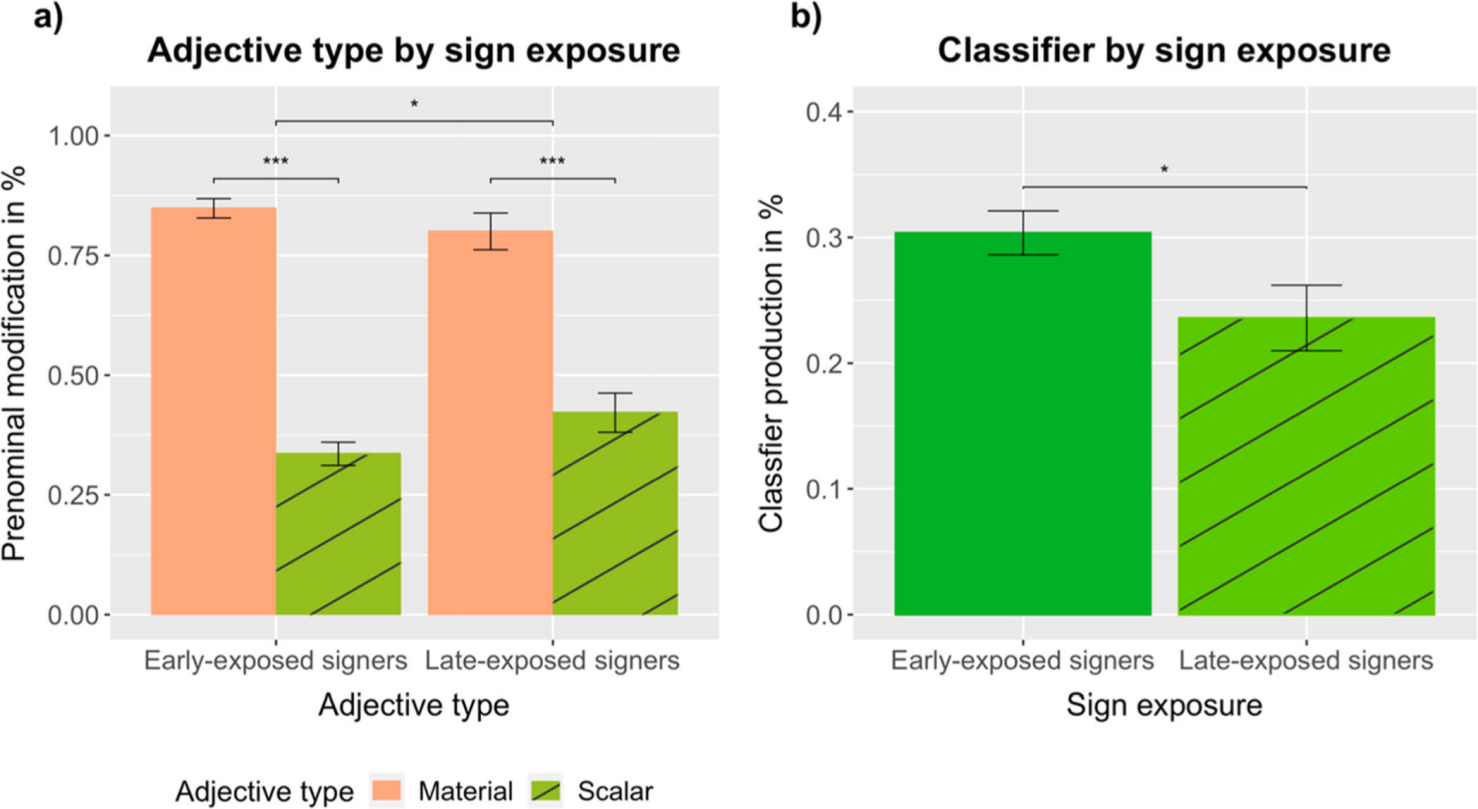
Percentage of (a) prenominal material and scalar adjectives and (b) classifiers (expressing scalar adjectives) produced by early-exposed and late-exposed ASL signers.

Adjective position was analyzed using a logistic mixed-effects model (prenominal = 1, postnominal = 0) with fixed effects of adjective type (material vs scalar) and sign exposure (early-exposed = 1, late-exposed = 0) and the maximal random effect structure for participants and items, including random intercepts for participants and items, plus random by-participant slopes for adjective type. Supporting our experimental predictions, there was a significant main effect of adjective type (*β* = −2.2007, SE = 0.5627, Z = −3.911, *p* < 0.0001), with material adjectives (*M* =.84, SD =.02) being produced in prenominal position significantly more often than scalar adjectives (*M* =.36, SD =.02) (see Fig. 6a; for video stills of produced word orders, see Fig. 7). There was also an adjective type by sign exposure interaction (*β* = −1.4919, SE = 0.6751, Z = −2.210, *p* < 0.0272), resulting from late-exposed ASL signers producing more scalar adjectives in prenominal position (*M* =.42, SD =.04) than early-exposed ASL signers (*M* =.34, SD =.02) (see Fig. 8a).

Since the difference between scalar and material adjectives could be driven by the use of classifiers in the scalar condition, we compared the position of just lexical signs in the material and scalar conditions. To this end, we analyzed lexical sign position using the same logistic mixed-effects model, which again revealed a significant main effect of adjective type (*β* = −1.6556, SE = 0.5307, Z = −3.119, *p* < 0.00182), confirming that lexical signs are encoded in prenominal position more frequently for material (*M* =.84, SD =.02) than for scalar properties (*M* =.52, SD =.03).

To investigate the effect of sign type on scalar adjective position, we analyzed the data from the scalar condition using a logistic mixed-effects model including fixed effects of sign type (classifier vs lexical sign) and sign exposure, random intercepts for participants and items, plus random by-participant slopes for sign type. Supporting our experimental predictions and replicating the results of Experiment 1, there was a significant main effect of sign type (*β* = 1.9759, SE = 0.5788, Z = 3.414, *p* < 0.00065), with lexical signs (*M* =.52, SD =.03) being produced in prenominal position significantly more often than classifiers (*M* =.22, SD =.03) (see Fig. 6b; for a visualization of the production rates of lexical signs and classifiers for individual scalar adjectives, see the Supplemental Material E).

To investigate the effect of sign exposure on the production of classifiers and lexical signs, we analyzed participants’ choice of sign type in the scalar condition (classifier = 1, lexical sign = 0) including a fixed effect of sign exposure and random intercepts for participants and items. As hypothesized, the results revealed a significant main effect of sign exposure (*β* = 1.0555, SE = 0.4115, Z = 2.565, *p* < 0.0104), with early-exposed ASL signers (*M* =.30, SD =.02) producing more classifiers than late-exposed ASL signers (*M* =.24, SD =.03) (see Fig. 8b).

#### Exploratory analyses of scalar adjectives in Experiment 1 and Experiment 2

The results of the scalar condition revealed that the ASL signers in Experiment 1 produced lexical signs in prenominal position 75% of the time, while the ASL signers in Experiment 2 did so 52% of the time (see Fig. 3b and Fig. 6b). Since the same scalar adjectives were tested in both experiments, we wanted to further explore this difference. The results of a logistic mixed-effects model revealed a significant main effect of sign type, with lexical signs being produced in prenominal position significantly more often than classifiers. There was also a significant main effect of Experiment, with signers in Experiment 1 producing more scalar adjectives in prenominal position than signers in Experiment 2 (for details, see Supplemental Material F).

A possible explanation for the different results observed in the scalar condition across Experiments 1 and 2 is that the color adjectives in Experiment 1 primed prenominal modification more strongly than the material adjectives in Experiment 2. To explore this possibility, we used the same logistic mixed-effects model to analyze scalar adjective position in blocks 1 and 2 separately (with block 2 being potentially sensitive to priming from block 1). The results from these analyses were inconclusive, however: the analysis of both blocks revealed a significant main effect of sign type and a marginally significant effect of Experiment (for details, see Supplemental Material F). Both individual differences (block 1) and priming from color adjectives (block 2) could therefore have an effect on scalar adjective position.

### Discussion of Experiment 2

The results from Experiment 2 replicated and extended the findings from Experiment 1. Signers produced prenominal material adjectives more frequently than they produced prenominal scalar adjectives, and this difference remained significant when we focused on the production of lexical signs. As predicted, classifiers were produced more often in postnominal position than lexical signs, and early-exposed ASL signers produced significantly more classifiers than late-exposed ASL signers when expressing scalar properties.

As in Experiment 1, we observed interesting differences in the production of different scalar adjectives, although their analysis is beyond the scope of this study (for a visualization of the production rates of lexical signs and classifiers for individual scalar adjectives, see the Supplemental Materials C and E). Future studies should investigate these differences, including the role of the noun in the expression of scalar properties in ASL.

Exploratory analyses across Experiments 1 and 2 confirmed that ASL signers’ tendency to encode scalar adjectives in prenominal position is mostly dependent on the type of sign (classifier vs lexical sign). However, scalar adjective position may also reveal individual preferences and be sensitive to priming from absolute adjectives, as suggested by the marginally significant effect of Experiment observed in trial blocks 1 and 2. Overall, the preference to encode classifiers in postnominal position seems to be more robust than the tendency to encode lexical signs for scalar properties in prenominal position (see Fig. 3b and Fig. 6b).

## Experiment 3

Given ASL signers’ preference to encode classifiers for scalar properties in postnominal position, in Experiment 3 we wanted to determine whether increasing the salience of the scalar adjective would lead to more prenominal scalar modification. To address this, we made size of the target an absolute property, using ‘pop-out’ displays where the target object was clearly larger or smaller than *all* the other objects in the display. According to the incremental efficiency hypothesis, making size an absolute property would render the corresponding scalar adjective more efficient in prenominal position because a comprehender could identify the intended referent by its size alone. Thus, the visual salience of the size contrast should elicit more prenominal modification in pop-out displays than in non-pop-out displays.

### Methods

#### Participants

The same participants who took part in Experiment 2 participated in Experiment 3.

#### Stimuli

**Fig. 9.**
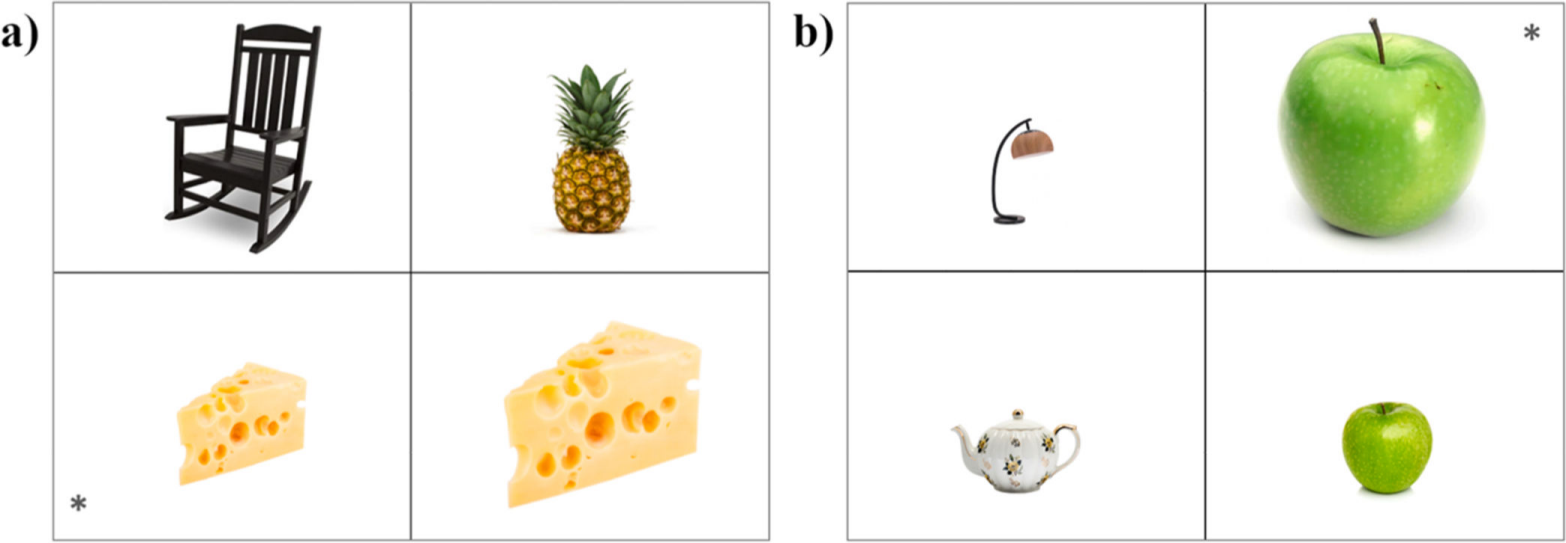
Sample items from the (a) non-pop-out condition (elicited adjective: ‘small’) and (b) the pop-out condition (elicited adjective: ‘big’). Targets are marked with an asterisk.

The linguistic materials only included the adjectives ‘big’ and ‘small’ because those were the scalar properties that were most amenable to a pop-out effect. Twelve visual displays were created for each adjective, following the same specifications in Experiments 1 and 2, for a total of 24 displays (see Supplemental Material G for the full list of stimuli). Half the displays created a pop-out effect such that the target object was considerably smaller or considerably bigger (depending on the adjective that was being elicited) than the other three objects in the display. In the other half of the displays, the relative size of the target object was comparable to Experiments 1 and 2, and did not create a pop-out effect (see Fig. 9 for sample displays). To avoid carryover effects, the pop-out and non-pop-out conditions were presented in two separate trial blocks, which were administered in two block orders (pop-out – non-pop-out and non-pop-out – pop-out). Participants were randomly allocated to one of the two lists of materials.

Participants were tested across two rounds of data collection. We adjusted the pictures after the first round, as follows: in the first testing session, the target and the contrast were different objects of the same kind. Because some of the items did not elicit size modification (e.g., participants referred to the target by its color), in the second testing session we used pictures of the same object for both the target and the contrast (just varying their relative size). As in Experiment 2, this change only affected the pictures that were shown: the target adjectives in each display were the same across testing sessions.

#### Procedure

Experiment 3 was run in the same session as Experiment 2. In this session, participants first completed Experiment 2, then participated in an unrelated eye-tracking task and then took part in Experiment 3. The experimental set up and procedure were the same as in Experiments 1 and 2. Stimuli were presented in two blocks of 12 trials with random assignment of participants to each list. Participants could take a break between blocks if needed.

#### Data processing

Data from Experiment 3 was processed following the same procedure as in Experiments 1 and 2. Again, Cohen’s Kappa (Cohen, 1960) was performed to determine coders agreement on adjective position and sign type. Coders exhibit strong agreement for adjective position (κ =.91, 95% CI.85,.97]) and strong agreement for sign type (κ =.79, 95% CI.70,.88]). Overall, we collected 1032 responses from our participants. As with Experiments 1 and 2, responses containing adjective position coded as *other* (396 responses) and sign type coded as *fingerspelled* (one response) were excluded, resulting in the exclusion of 397 (36.8%) responses.

#### Analysis plan and predictions

Adjective position (prenominal vs postnominal) was analyzed using a logistic mixed-effects model that tested the effect of condition (pop-out vs non-pop-out). According to the incremental efficiency hypothesis, pop-out displays should elicit more prenominal size modification than non-pop-out displays. Our second prediction was that classifiers would be more likely to be encoded in postnominal position. To test this hypothesis, we analyzed adjective position using a logistic mixed-effects model testing for the effect of sign type (classifier vs lexical sign). Finally, we explored the use of classifiers by early-exposed and late-exposed ASL signers, for which we analyzed the production of classifiers and lexical signs using a logistic mixed-effects model testing for the effect of sign exposure (early-exposed vs late-exposed).

### Results

**Fig. 10.**
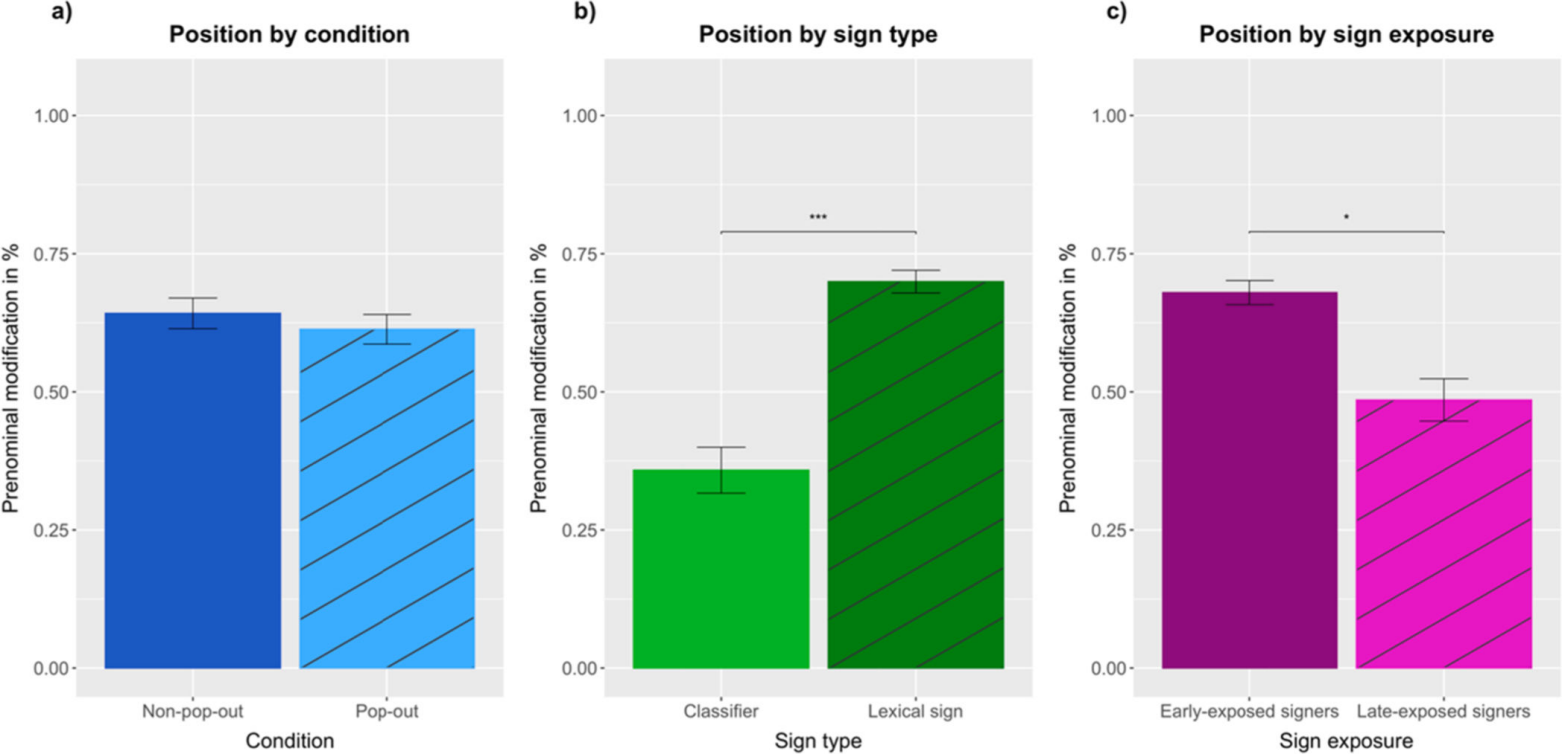
Percentage of (a) prenominal size modification in the non-pop-out and pop-out conditions, (b) prenominal classifier and lexical signs and (c) prenominal adjectives produced by early-exposed and late-exposed ASL signers.

As in Experiments 1 and 2, adjectives ‘big’ and ‘small’ were produced as lexical signs around 75% of the time. To investigate the effect of the pop-out manipulation, we analyzed adjective position using a logistic mixed-effects model (prenominal = 1, postnominal = 0) with fixed effects of condition (pop-out vs non-pop-out) and sign exposure (early-exposed = 1, late-exposed = 0) and the maximal random effect structure for participants and items, including random intercepts for participants and items, plus random by-participant slopes for condition. This model only revealed a marginally significant effect of sign exposure (*β* = 1.5842, SE = 0.9352, Z = 1.694, *p* = 0.0903), with early-exposed ASL signers (*M* =.68, SD =.02) producing more prenominal size modification than late-exposed ASL signers (*M* =.49, SD =.04). The effect of condition was non-significant (*β* = 0.4458, SE = 0.5888, Z = 0.757, *p* = 0.4490; see Fig. 10a).

To investigate the effect of sign type on size adjective position, we used a logistic mixed-effects model including fixed effects of sign type (classifier vs lexical sign) and sign exposure (early-exposed vs late-exposed), random intercepts for participants and items, plus random by-participant slopes for sign type. Supporting our experimental predictions, there was a significant main effect of sign type (*β* = 1.8165, SE = 0.6988, Z = 2.599, *p* < 0.0094), with lexical signs (*M* =.70, SD =.02) being produced in prenominal position significantly more often than classifiers (*M* =.36, SD =.04) (see Fig. 10b; for a visualization of the production rates of lexical signs and classifiers for individual scalar adjectives, see the Supplemental Material H). There was also a significant main effect of sign exposure (*β* = 2.0978, SE = 1.0602, Z = 1.979, *p* < 0.0479), with early-exposed ASL signers (*M* =.68, SD =.02) producing more prenominal size modification than late-exposed ASL signers (*M* =.49, SD =.04) (see Fig. 10c).

To investigate the effect of sign exposure on the production of classifiers and lexical signs, we analyzed participants’ choice of sign type (classifier = 1, lexical sign = 0) using a logistic mixed-effects model including a fixed effect of sign exposure and random intercepts for participants and items. Contrary to our predictions, the effect of sign exposure was not significant (*β* = −0.3956, SE = 0.9203, Z = −0.430, *p* = 0.6673).

#### Exploratory analyses of ‘big’ and ‘small’ across Experiment 2 and Experiment 3

Even though the same ASL signers took part in Experiments 2 and 3, age of sign exposure had different effects in the two experiments. In Experiment 2, early-exposed ASL signers produced more postnominal scalar adjectives than late-exposed ASL signers, while in Experiment 3, early-exposed signers produced more prenominal size adjectives than late-exposed signers. In addition, early-exposed ASL signers produced more classifiers than late-exposed ASL signers in Experiment 2, while there was no difference between the two groups in Experiment 3. These patterns of results suggest that there was more variability in adjective position and sign type in Experiment 2 than Experiment 3, likely due to the greater variety of adjectives that were elicited.

Previous reference production studies with English and Spanish speakers (Rubio-Fernandez, 2016, 2019) as well as Dutch speakers (Tarenskeen et al., 2015) reported that participants adopted referential strategies early on (e.g., whether or not to use color adjectives) and used the same type of referential expression until the end of the task. To explore whether participants in Experiment 3 had also used prenominal size modification consistently (potentially masking any effect of the pop-out manipulation), we used the production of ‘big’ and ‘small’ in Experiment 2 as a post-hoc baseline, and compared it with the two visual conditions in Experiment 3. We carried out separate analyses for block 1 and block 2 to explore different questions.

Analysis of the first trial block allows us to control for any priming effects from a previous block. Thus, in the first trial block of Experiment 2, participants in the scalar condition had not yet been exposed to (and potentially primed by) the material condition (which elicited high rates of prenominal modification). Likewise, in the first block of Experiment 3, participants in the pop-out condition would not have been exposed to the non-pop-out trials yet. The results of a logistic mixed-effects model revealed a significant difference between Experiment 2 and the pop-out condition in Experiment 3, with higher rates of prenominal size modification being observed in the pop-out condition. Importantly, the difference between Experiment 2 and the non-pop-out condition in Experiment 3 was not significant (for details, see Supplemental Material I). Thus, in the first trial block, participants in Experiment 3 produced more prenominal size modification in the pop-out condition than participants in Experiment 2 (who were presented with non-pop-out displays and used a greater variety of scalar adjectives). By contrast, the rates of prenominal size modification were comparable between Experiment 2 and the non-pop-out condition in Experiment 3.

An analysis of the second trial block of Experiments 2 and 3 could potentially reveal the degree to which participants were susceptible to priming from the first trial block. We therefore conducted the same logistic mixed-effects model on the data from block 2, and observed two condition by sign exposure interactions. The first significant interaction was between Experiment 2 and the pop-out condition in Experiment 3, with early-exposed ASL signers producing more prenominal size modification in the pop-out condition than in Experiment 2, while the reverse pattern was observed for late-exposed ASL signers. The second significant interaction was between Experiment 2 and the non-pop-out condition in Experiment 3, with early-exposed ASL signers producing more prenominal size modification in the non-pop-out condition in Experiment 3, while late-exposed ASL signers produced comparable rates of prenominal modification in the two conditions (for details, see Supplemental Material I). This pattern suggests that early-exposed signers were more consistent (or prone to ‘self-priming’) than late-exposed signers across the two trial blocks in Experiment 3.

### Discussion of Experiment 3

The results of Experiment 3 did not show more prenominal size modification in the pop-out condition than in the non-pop-out condition, contrary to the predictions of the incremental efficiency hypothesis. As in the first two experiments, ‘big’ and ‘small’ were expressed as lexical signs 75% of the time, and lexical signs were produced in prenominal position more often than classifiers. Given that ASL signers prefer to encode ‘big’ and ‘small’ in prenominal position, even without a pop-out manipulation, we recognize that the pop-out size manipulation was a hard test of the incremental efficiency hypothesis. However, we considered that making size an absolute property was visually ‘easier’ than with other scalar properties such as width or thinness (which were more often expressed in postnominal position, but are harder to make salient as absolute properties). This was our rationale for using a pop-out size manipulation in Experiment 3.

Interestingly, early-exposed ASL signers produced more prenominal size modification than late-exposed ASL signers. We hypothesized that the ASL signers who participated in Experiments 2 and 3 revealed a higher degree of response consistency in the latter experiment because it only elicited two adjectives. We investigated that possibility in two exploratory analyses comparing the use of ‘big’ and ‘small’ in Experiments 2 and 3. Our results support the predictions of the incremental efficiency hypothesis, although they should be interpreted with caution given the exploratory nature of the analyses.

In the second trial block, early-exposed and late-exposed ASL signers showed contrasting patterns. We interpret these results as revealing differences in response consistency between the two groups.

## General discussion

Unlike many spoken languages, ASL has relatively flexible adjective position, allowing both prenominal and postnominal modification, with certain restrictions (see Bernath, 2010; Loos, 2022; Valli & Lucas, 2000). ASL therefore offers a natural test of the relative efficiency of adjective position for referential communication, which until now had only been tested in spoken languages with prenominal or postnominal modification (Rubio-Fernandez, 2016, 2019, 2021; Rubio-Fernandez & Jara-Ettinger, 2020; Rubio-Fernandez et al., 2020; Wu & Gibson, 2021; Kachakeche et al., 2021). By testing the incremental efficiency hypothesis in ASL, we were able to further investigate whether referential efficiency may be a general principle that applies across languages and modalities.

Our investigation focused on the effects of three factors on adjective placement; namely, adjective semantics (absolute vs scalar), sign type (lexical sign vs classifier) and age of sign exposure (early vs late). According to the incremental efficiency hypothesis, ASL signers, like speakers of spoken languages, should produce efficient referential expressions that are sensitive to adjective position. Thus, we predicted that ASL signers would produce prenominal modification when expressing absolute properties such as color or material, which can be interpreted ahead of the noun, thus allowing the comprehender to efficiently use that information (e.g., identifying the referent by its color). By contrast, we predicted that scalar adjectives such as size or width, which can only be interpreted relative to the noun they modify, would be more efficient when encoded in postnominal position (for related evidence with several spoken languages, see Jara-Ettinger & Rubio-Fernandez, 2021; Rubio-Fernandez & Jara-Ettinger, 2020; Rubio-Fernandez et al., 2020).

The results of two referential communication tasks partially supported our predictions, revealing high rates of prenominal modification for both color (Experiment 1) and material adjectives (Experiment 2). Scalar adjectives, on the other hand, were encoded both pre- and postnominally, with this distinction being determined mainly by the type of sign that was employed: lexical signs were used more often in prenominal position, while classifiers were used more often in postnominal position. Further supporting the incremental efficiency hypothesis, analyses of lexical sign position (i.e. excluding classifiers) revealed, in both experiments, that lexical signs for color and material properties were produced significantly more often prenominally than lexical signs for scalar properties.

In Experiment 3, we used a pop-out manipulation that made the target size an absolute property in order to probe higher rates of prenominal scalar modification. This was a hard test of the incremental efficiency hypothesis, since signers in Experiments 1 and 2 had already shown a preference to produce prenominal size adjectives. Indeed, ‘big’ and ‘small’ were not encoded more often in prenominal position in the pop-out condition, with signers being consistent in their prenominal preference regardless of display type. However, the results of an exploratory analysis across Experiments 2 and 3 revealed that the pop-out condition did elicit more prenominal size modification than the non-pop-out condition in Experiment 2, while the non-pop-out displays elicited comparable rates of prenominal size adjectives in both experiments. Future studies should further investigate ASL signers’ sensitivity to visual salience manipulations when expressing scalar properties that are normally expressed in postnominal position (unlike the size dimension investigated here).

Overall, the results of our experiments offered support for the incremental efficiency hypothesis. ASL signers in our study utilized the flexibility in their adjective placement to make efficient choices that could facilitate processing in real-time referential communication. It must be noted, however, that ASL reference production is not only determined by adjective semantics and efficiency considerations. Sign type and age of ASL exposure also played significant roles in our study and were likely at least partially driving the observed patterns, deepening our understanding of referential communication as a complex process motivated by multiple constraints.

### Classifier use in expressing scalar properties

When asked to describe a referent object that contrasted in scale with another object, signers most frequently expressed the scalar property using a lexical adjective sign. Across all three experiments, signers used classifiers to express scalar properties about a quarter of the time, although this varied by experiment, by type of scalar adjective, and by the linguistic background of the signer. When signers did choose to use classifiers, they were produced in postnominal position significantly more often than lexical signs. This pattern matches our predictions based on the semantic structure of classifier constructions (Tang, Li, & He, 2021). Since classifiers (specifically SASS constructions, as used here) incorporate relative properties of the referent noun, it is more referentially transparent to first introduce the referent noun, and then to incorporate features of that noun in the classifier (Barberà & Quer, 2018). Notably, we also observed instances in which signers chose to produce a single complex classifier construction without first introducing the referent noun and without using lexical signs for either the adjective or noun. Such constructions were not included in our analysis, as our primary interest was in adjective position; however, the presence of these complex constructions provides further evidence for the wide range of possible constructions to express a single concept in sign languages (Frederiksen & Mayberry, 2016; Hodge et al., 2019).

### Linguistic experience effects on ASL production

Delayed exposure to a sign language as a first language is known to have myriad effects on adult sign proficiency and processing (Mayberry & Kluender, 2018; Krebs et al., 2020). Our data revealed that age of ASL exposure also affects how signers express adjectives in a referential communication task. Specifically, in Experiment 2, late-exposed signers were more likely than early-exposed signers to produce scalar adjectives in prenominal position; this preference was largely driven by the fact that late-exposed signers were more likely than early-exposed signers to use lexical signs vs classifier constructions.

A preference for lexical signs among late-exposed signers may arise directly from their lack of early exposure to a full and accessible language. Children acquiring ASL show a protracted timeline for the acquisition of classifiers (Kantor 1982; Schick 1990). Native-signing children begin using classifier constructions at three or four years of age, but may omit or produce errors in their productions (Schick, 1990). Morgan and Woll (2003) found that 4- to 13-year-old children acquiring British Sign Language (BSL) differed from adults in their use of entity classifiers as a form of reference, particularly when maintaining reference in discourse. Thus, although the developmental milestones in classifier acquisition are not fully described, it is clear that full mastery of the classifier system in native or early-exposed learners of a sign language is a protracted process. As such, individuals who were not exposed to ASL until after early childhood may have delayed or incomplete acquisition of the full classifier system. It is also possible that the participants who acquired ASL later were influenced by English to a greater extent than those who acquired ASL at birth. While deaf adults who use ASL are generally bilingual in that they use both ASL and (spoken and/or written) English, it is possible that English was the more dominant language in childhood for signers who did not learn ASL until later.

The effects of sign exposure were not fully consistent across all manipulations in the current study. In Experiment 3, early-exposed signers used more prenominal adjectives than late-exposed signers, and there were no significant group differences in the frequency of classifier constructions in Experiment 3. As the non-pop-out condition did not change across experiments, it is likely that the increased use of prenominal adjectives arose from the presence of the pop-out condition. Specifically, since the pop-out condition led to increased use of prenominal adjectives, we speculate that early-exposed signers were more likely to experience a priming effect, wherein the salience of the scalar property in the pop-out condition primed an increased use of prenominal adjectives in the non-pop-out condition. Early-exposed signers may have been more susceptible to this priming effect because they engage in more efficient and automatic processing than late-exposed signers (Emmorey et al., 1995; Mayberry & Fisher, 1989), or because they have greater overall proficiency in ASL (Bernolet et al., 2013).

### Natural word orders

Referential efficiency is one factor driving word order, but is not the only one. An alternative proposal has been tested in silent gesture experiments, in which hearing participants communicate using their hands and no speech. These studies have been used to investigate whether there is a basic or ‘natural’ word order in human languages (e.g., Goldin-Meadow et al., 2008). In a recent study, Culbertson et al. (2020) used the silent gesture paradigm to investigate whether there is a natural ordering preference for nominal modifiers: demonstratives, numerals, and adjectives. While their focus was on the relative ordering of multiple modifiers rather than their position with respect to the noun, their results showed a clear overall preference for post-nominal adjectives (despite the fact that the participants in the experiment were native English speakers). This result appears to match typological data from Dryer (2013), who reviewed adjective ordering across over 1000 languages, and observed that more than twice as many languages in his sample exhibit dominant postnominal adjective order than exhibit dominant prenominal adjective order (879 vs. 373 languages, respectively).

The present results do not match the natural word order observed by Culbertson et al. (2020) using the silent gesture paradigm, revealing instead different preferences depending on adjective semantics and sign type. Crucially, however, the stimuli used by Culbertson et al. were carefully designed to rule out any efficiency considerations, with all aspects of what was being conveyed by the gestures being equally informative. Additionally, unlike the spontaneous gestures produced by participants in Culbertson et al. (2020), ASL and other sign languages evolved over multiple generations of signers who are using the language in communicative interaction. Therefore, the results of the two studies may not be incompatible but simply reveal different word order preferences under very different experimental conditions. Future studies should investigate how the naturalness of different word orders interacts with communicative efficiency constraints.

## Conclusions and future directions

The results of our study confirm ASL signers produce more prenominal modification when expressing absolute properties, such as color or material, than when expressing scalar properties, such as size. These findings are consistent with the incremental efficiency hypothesis, confirming that language structure can be exploited for communicative efficiency (Gibson et al., 2019). Future studies should investigate whether adjective use is also efficient when referring to absent entities in conversation. It is possible that referring to ‘the tall man’ is efficient in a crowd of people, for example, but referring to the same person as ‘the angry man’ may be a more efficient description for memory retrieval (e. g., if your interlocutor had an unpleasant exchange with this man). Therefore, the efficiency of adjective choice for memory retrieval would offer a further and interesting test of the communicative efficiency hypothesis (for discussion, see Jara-Ettinger & Rubio-Fernandez, 2021).

Another robust finding that was observed across the three experiments in this study is that scalar properties tend to be encoded in prenominal position when expressed as lexical signs, while they are encoded in postnominal position when expressed as classifiers. In addition, age of ASL exposure also affected referential choice, with early signers producing higher rates of classifiers than late signers. The picture emerging from this study is therefore a complex one, calling for further research on referential communication in ASL and the different linguistic, pragmatic and developmental factors affecting referential choice across modalities.

## Supplementary Material

supplementary material

## Footnotes

1 Notational conventions: signs are glossed in small caps. A hyphen between signs indicates that more than one word is needed to gloss a single sign.

2 We use the term ‘classifier’ herein to refer to the subset of classifiers observed in the current study, which are predominantly SASS constructions.

3 We also documented participants’ parental hearing status. Participants had deaf parents (n = 20) or hearing parents (n = 22), or did not report (n = 9). Analysis by parental hearing status yielded almost identical patterns in analysis as age of ASL exposure, thus we decided to use age of exposure as a more informative grouping variable.
