# Supplementary material for: Adjective position and referential efficiency in American Sign Language: Effects of adjective semantics, sign type and age of sign exposure
Source: J Mem Lang. Author manuscript; Available in PMC 2024 Apr 25. (PMC11044888; doi:10.1016/j.jml.2022.104348)
Supplement: supplementary material [file NIHMS1944845-supplement-supplementary_material.docx]

**SUPPLEMENTAL MATERIAL**

**Appendix A:** full stimuli list for Experiment 1

| **Adjective class/type** | **Adjective** | **Noun** | **Target** |
| --- | --- | --- | --- |
| color | BLACK | FEATHER | BLACK FEATHER |
| color | BLACK | DRUM | BLACK DRUM |
| color | BLACK | LAMP | BLACK LAMP |
| color | BLUE | PILLOW | BLUE PILLOW |
| color | BLUE | BUS | BLUE BUS |
| color | BLUE | BUCKET | BLUE BUCKET |
| color | BROWN | BELT | BROWN BELT |
| color | BROWN | SUITCASE | BROWN SUITCASE |
| color | BROWN | PANTS | BROWN PANTS |
| color | GREEN | LEAF | GREEN LEAF |
| color | GREEN | MUG | GREEN MUG |
| color | GREEN | BOOK | GREEN NOTEBOOK |
| color | ORANGE | WATCH | ORANGE WATCH |
| color | ORANGE | BUTTERFLY | ORANGE BUTTERFLY |
| color | ORANGE | SKIRT | ORANGE SKIRT |
| color | RED | HAT | RED HAT |
| color | RED | FISH | RED FISH |
| color | RED | CAR | RED CAR |
| color | WHITE | DOG | WHITE DOG |
| color | WHITE | FLOWER | WHITE FLOWER |
| color | WHITE | MUG | WHITE MUG |
| color | YELLOW | SHIRT | YELLOW SHIRT |
| color | YELLOW | UMBRELLA | YELLOW UMBRELLA |
| color | YELLOW | BIKE | YELLOW BIKE |
| scalar | BIG | RABBIT | BIG RABBIT |
| scalar | BIG | WASHING MACHINE | BIG WASHING MACHINE |
| scalar | BIG | BALL | BIG BALL |
| scalar | NARROW | TAPE | NARROW TAPE |
| scalar | NARROW | TIE | NARROW TIE |
| scalar | SHORT | RULER | SHORT RULER |
| scalar | SHORT | CANDLE | SHORT CANDLE |
| scalar | SHORT | PENCIL | SHORT PENCIL |
| scalar | SMALL | TOWEL | SMALL TOWEL |
| scalar | SMALL | BIRD | SMALL BIRD |
| scalar | SMALL | BRUSH | SMALL BRUSH |
| scalar | TALL | GLASS | TALL GLASS |
| scalar | TALL | BUILDING | TALL BUILDING |
| scalar | TALL | TREE | TALL TREE |
| scalar | THICK | GLASSES | THICK GLASSES |
| scalar | THICK | SOCKS | THICK SOCKS |
| scalar | THICK | BOOK | THICK BOOK |
| scalar | THIN | PASTA | THIN PASTA |
| scalar | THIN | MATTRESS | THIN MATTRESS |
| scalar | THIN | TIRE | THIN TIRE |
| scalar | THIN | PIZZA | THIN PIZZA |
| scalar | WIDE | WINDOW | WIDE WINDOW |
| scalar | WIDE | DOOR | WIDE DOOR |
| scalar | WIDE | COUCH | WIDE COUCH |

**Appendix B**: Breakdown of adjective position category ‘other’

Adjective position was coded as *prenominal*, *postnominal* or *other*. The category *other* included instances where either (a) only the adjective was used, (b) the adjective or the noun occurred twice (e.g., ball big ball or big ball big), (c) the adjective was incorporated in the noun using classifiers (i.e., adjective and noun are expressed as one sign; e.g., classifier[big-ball]), (d) a different adjective type was used (e.g., a color adjective instead of the intended scalar adjective), or (e) no adjective was produced at all. The tables below present a breakdown of the category *other* for Experiment 1 (Table A), Experiment 2 (Table B) and Experiment 3 (Table 3). All values are in given in percent.

Table A. Overview for Experiment 1.

|  | Color | Scalar |
| --- | --- | --- |
| (a) Adjective only | 0.3 % | 0 % |
| (b) Noun or adjective doubled | 1.5 % | 8.6 % |
| (c) Incorporation | 0 % | 2.1 % |
| (d) Unintended adjective | 3.0 % | 70.0 % |
| (e) No adjective | 1.8 % | 12.7 % |

Table B. Overview for Experiment 2.

|  | Material | Scalar |
| --- | --- | --- |
| (a) Adjective only | 0 % | 0.5 % |
| (b) Noun or adjective doubled | 3.6 % | 4.8 % |
| (c) Incorporation | 0 % | 0 % |
| (d) Unintended adjective | 43.7 % | 39.2 % |
| (e) No adjective | 3.3 % | 4.9 % |

Table C. Overview for Experiment 3.

|  | Pop-out | Non-popout |
| --- | --- | --- |
| (a) Adjective only | 0.8 % | 0 % |
| (b) Noun or adjective doubled | 1.3 % | 3.8 % |
| (c) Incorporation | 0 % | 0 % |
| (d) Unintended adjective | 40.4 % | 47.9 % |
| (e) No adjective | 3.3 % | 2.5 % |

**Appendix C**: Production of sign types (lexical sign and classifiers) for individual scalar adjectives in Experiment 1


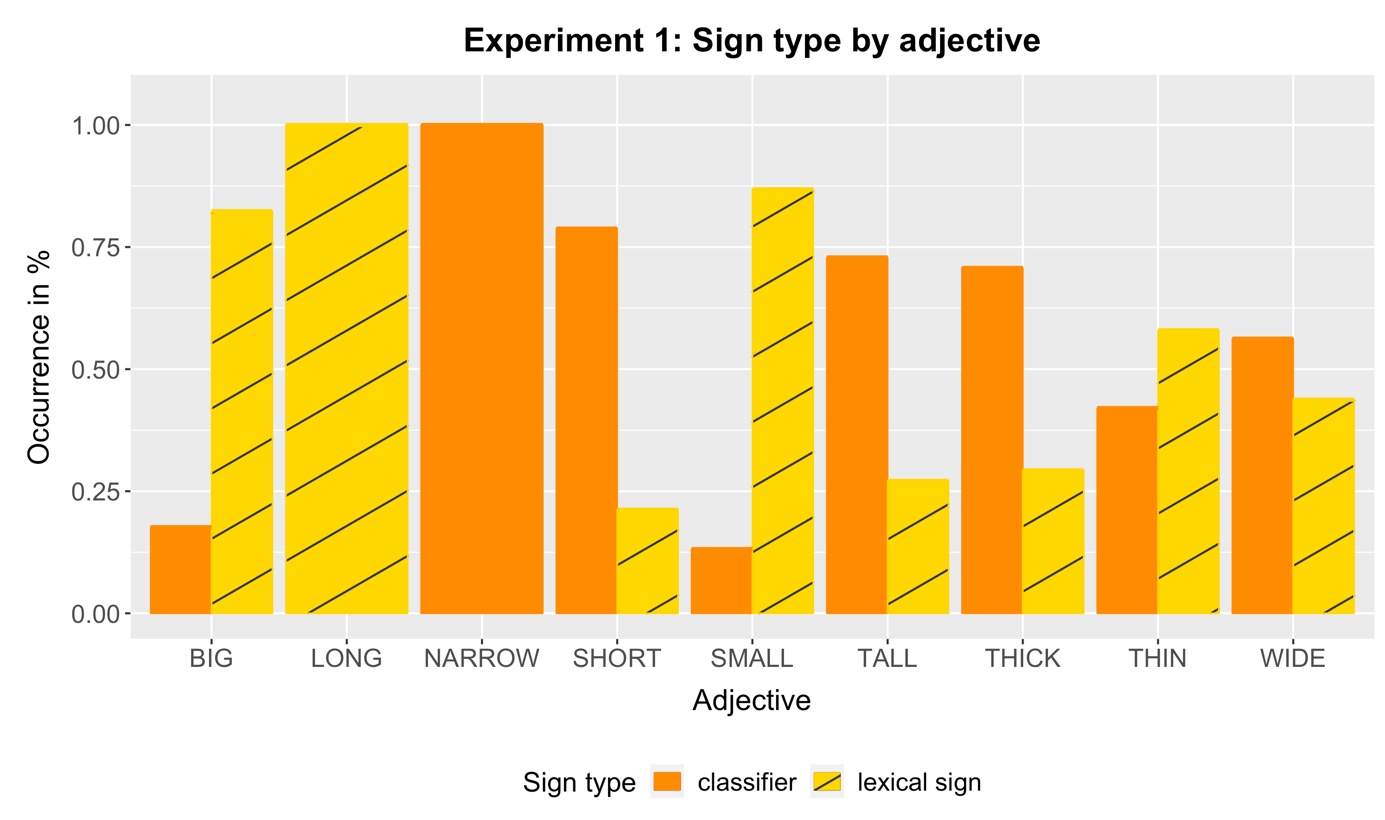


**Appendix D**: full stimuli list for Experiment 2

| **Adjective class/type** | **Adjective** | **Noun** | **Target** |
| --- | --- | --- | --- |
| material | COTTON | SHOE | COTTON SHOE |
| material | COTTON | SHIRT | COTTON SHIRT |
| material | COTTON | DRESS | COTTON DRESS |
| material | GLASS | BOWL | GLASS BOWL |
| material | GLASS | BOTTLE | GLASS BOTTLE |
| material | GLASS | VASE | GLASS VASE |
| material | GOLD | EARRINGS | GOLD EARRINGS |
| material | GOLD | RING | GOLD RING |
| material | GOLD | NECKLACE | GOLD NECKLACE |
| material | LEATHER | BAG | LEATHER BAG |
| material | LEATHER | WALLET | LEATHER WALLET |
| material | LEATHER | SHOES | LEATHER SHOES |
| material | METAL | FORK | METAL FORK |
| material | METAL | WATERING CAN | METAL WATERING CAN |
| material | METAL | HAMMER | METAL HAMMER |
| material | PAPER | ENVELOPE | PAPER ENVELOPE |
| material | PAPER | NAPKIN | PAPER NAPKIN |
| material | PAPER | CUP | PAPER CUP |
| material | PLASTIC | MUG | PLASTIC MUG |
| material | PLASTIC | BOAT | PLASTIC BOAT |
| material | PLASTIC | PLATE | PLASTIC PLATE |
| material | WOOD | SPOON | WOODEN SPOON |
| material | WOOD | TABLE | WOODEN TABLE |
| material | WOOD | BOX | WOODEN BOX |
| scalar | BIG | RABBIT | BIG RABBIT |
| scalar | BIG | WASHING MACHINE | BIG WASHING MACHINE |
| scalar | BIG | BALL | BIG BALL |
| scalar | NARROW | PANTS | NARROW PANTS |
| scalar | NARROW | RUG | NARROW RUG |
| scalar | NARROW | TIE | NARROW TIE |
| scalar | NARROW | TIRE | NARROW TIRE |
| scalar | SHORT | RULER | SHORT RULER |
| scalar | SHORT | CANDLE | SHORT CANDLE |
| scalar | SHORT | PENCIL | SHORT PENCIL |
| scalar | SMALL | TOWEL | SMALL TOWEL |
| scalar | SMALL | BIRD | SMALL BIRD |
| scalar | SMALL | BRUSH | SMALL BRUSH |
| scalar | TALL | GLASS | TALL GLASS |
| scalar | TALL | BUILDING | TALL BUILDING |
| scalar | TALL | TREE | TALL TREE |
| scalar | THICK | GLASSES | THICK GLASSES |
| scalar | THICK | SOCKS | THICK SOCKS |
| scalar | THICK | BOOK | THICK BOOK |
| scalar | THIN | MATTRESS | THIN MATTRESS |
| scalar | THIN | PIZZA | THIN PIZZA |
| scalar | WIDE | WINDOW | WIDE WINDOW |
| scalar | WIDE | DOOR | WIDE DOOR |
| scalar | WIDE | SCREEN | WIDE SCREEN |

**Appendix E**: Production of sign types (lexical sign and classifiers) for individual scalar adjectives in Experiment 2


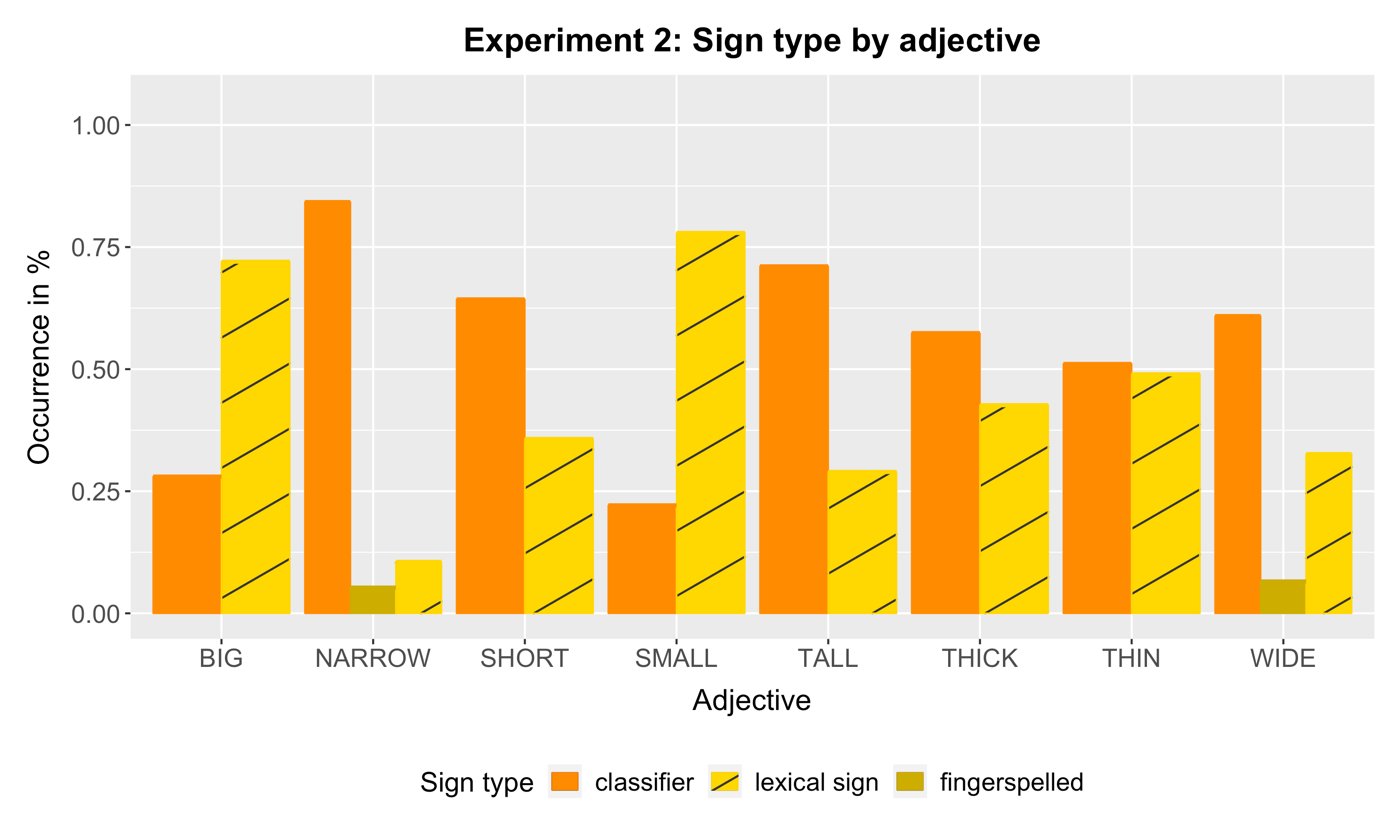


**Appendix F**: Exploratory analyses of scalar adjectives in Experiment 1 and Experiment 2

We analyzed scalar adjective position in Experiment 1 and Experiment 2 (prenominal=1, postnominal=0) using a logistic mixed-effects model including fixed effects of sign type (classifier vs lexical sign) and Experiment (E1 vs E2), random intercepts for participants and items plus random by-participant slopes for sign type. The results revealed a significant main effect of sign type (*β*=2.0707, SE=0.3481, Z=5.949, *p*<0.0001), with lexical signs being produced in prenominal position significantly more often than classifiers. There was also a significant main effect of Experiment (*β*=-1.5056, SE=0.6105, Z=-2.466, *p*<0.0138), with signers in Experiment 1 producing more scalar adjectives in prenominal position than signers in Experiment 2.

It might be possible that the color adjectives in Experiment 1 primed prenominal modification more strongly than the material adjectives in Experiment 2. To explore this possibility, we used the same logistic mixed-effects model to analyze scalar adjective position in blocks 1 and 2 separately (with block 2 being potentially sensitive to priming from block 1). The results from the block 1 analysis revealed a significant main effect of sign type (*β*=1.7037, SE=0.4440, Z=3.837, *p*<0.00013), and a marginally significant effect of Experiment (*β*=-1.3037, SE=0.7753, Z=-1.681, *p*<0.09267). The results from the block 2 analysis also revealed a significant main effect of sign type (*β*=2.0263, SE=0.5582, Z=3.630, *p*<0.00029), and a marginally significant effect of Experiment (*β*=-1.8988, SE=0.9788, Z=-1.940, *p*<0.05239).

**Appendix G**: full stimuli list for Experiment 3

| **Condition** | **Adjective** | **Noun** | **Target** |
| --- | --- | --- | --- |
| non-pop-out | BIG | BICYCLE | BIG BICYCLE |
| non-pop-out | BIG | CAT | BIG CAT |
| non-pop-out | BIG | TURTLE | BIG TURTLE |
| non-pop-out | BIG | ICECREAM | BIG ICECREAM |
| non-pop-out | BIG | SCISSORS | BIG SCISSORS |
| non-pop-out | BIG | OWL | BIG OWL |
| non-pop-out | SMALL | CHEESE | SMALL CHEESE |
| non-pop-out | SMALL | BIRD | SMALL BIRD |
| non-pop-out | SMALL | GRAPES | SMALL GRAPES |
| non-pop-out | SMALL | COOKIE | SMALL COOKIE |
| non-pop-out | SMALL | BEAR | SMALL BEAR |
| non-pop-out | SMALL | BUG | SMALL BUG |
| pop-out | BIG | BUTTERFLY | BIG BUTTERFLY |
| pop-out | BIG | TRAIN | BIG TRAIN |
| pop-out | BIG | APPLE | BIG APPLE |
| pop-out | BIG | KEY | BIG KEY |
| pop-out | BIG | FLOWER | BIG FLOWER |
| pop-out | BIG | ORANGE | BIG ORANGE |
| pop-out | SMALL | GLOVES | SMALL GLOVES |
| pop-out | SMALL | SHEEP | SMALL SHEEP |
| pop-out | SMALL | PAINTBRUSH | SMALL PAINTBRUSH |
| pop-out | SMALL | CHAIR | SMALL CHAIR |
| pop-out | SMALL | SCREWDRIVER | SMALL SCREWDRIVER |
| pop-out | SMALL | HAMBURGER | SMALL HAMBURGER |

**Appendix H**: Production of sign types (lexical sign and classifiers) for individual scalar adjectives in Experiment 3


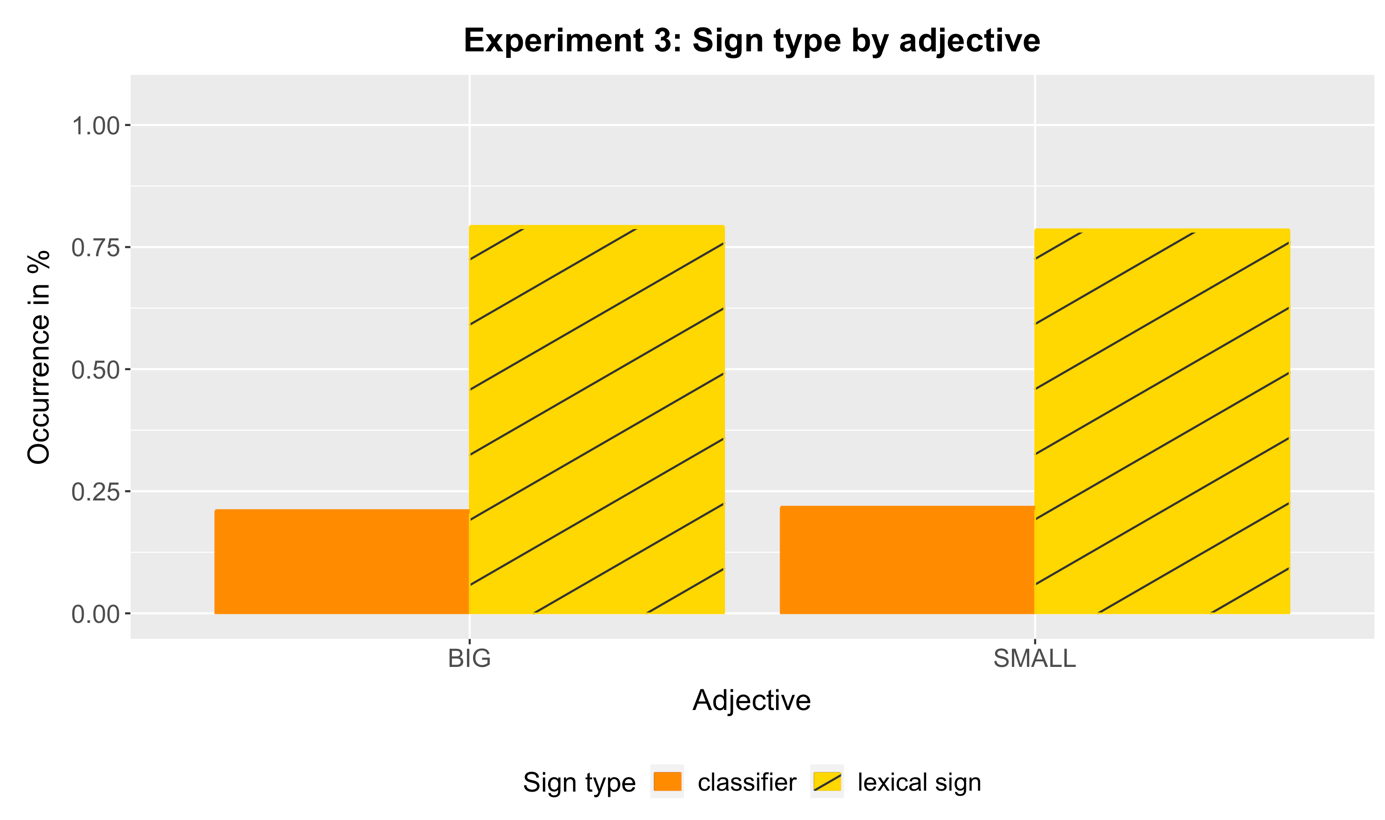


**Appendix I**: Exploratory analyses of ‘big’ and ‘small’ across Experiment 2 and Experiment 3

In the first trial block of Experiment 2, participants in the scalar condition had not yet been exposed to (and potentially primed by) the material condition (which elicited high rates of prenominal modification). Likewise, in the first block of Experiment 3, participants in the pop-out condition would not have been exposed to the non-pop-out trials yet. The results of a logistic mixed-effects model with fixed effects of condition (Experiment 2, Experiment 3/ pop-out, Experiment 3/ non-pop-out) and random intercepts for participants and items revealed a significant difference between Experiment 2 and the pop-out condition in Experiment 3 (*β*=1.9381, SE=0.9009, Z=2.151, *p*<0.0315), with higher rates of prenominal size modification being observed in the pop-out condition. Importantly, the difference between Experiment 2 and the non-pop-out condition in Experiment 3 was not significant (*β*=-0.4733, SE=0.8870, Z=-0.534, *p*=0.5936).

An analysis of the second trial block of Experiments 2 and 3 could potentially reveal the degree to which participants were susceptible to priming from the first trial block. We therefore conducted the same logistic mixed-effects model on the data from block 2, and observed two condition by sign exposure interactions. The first significant interaction was between Experiment 2 and the pop-out condition in Experiment 3 (*β*=2.3161, SE=0.9568, Z=2.421, *p*<0.0156), with early ASL signers producing more prenominal size modification in the pop-out condition than in Experiment 2, while the reverse pattern was observed for late ASL signers. The second significant interaction was between Experiment 2 and the non-pop-out condition in Experiment 3 (*β*=3.5030, SE=1.3752, Z=2.547, *p*<0.0110), with early ASL signers producing more prenominal size modification in the non-pop-out condition in Experiment 3, while late ASL signers produced comparable rates of prenominal modification in the two conditions.
